# Isolation, characterization and application of bacteriophage PSDA-2 against *Salmonella* Typhimurium in chilled mutton

**DOI:** 10.1371/journal.pone.0262946

**Published:** 2022-01-24

**Authors:** Ziyu Sun, Mandlaa, Hui Wen, Li Ma, Zhongjun Chen

**Affiliations:** 1 College of Food Science and Engineering, Inner Mongolia Agricultural University, Hohhot, China; 2 College of Life Sciences, Inner Mongolia Agricultural University, Hohhot, China; Nitte University, INDIA

## Abstract

*Salmonella* is a common foodborne pathogen, especially in meat and meat products. Lytic phages are promising alternatives to conventional methods for *Salmonella* biocontrol in food and food processing. In this study, a virulent bacteriophage (PSDA-2) against *Salmonella enterica* serovar Typhimurium was isolated from the sewage and it was found that PSDA-2 belongs to *Cornellvirus* genus of *Siphoviridae* family by morphological and phylogenetic analysis. Based on the one-step growth curve, PSDA-2 has a short latent period (10 min) and a high burst size (120 PFU/cell). The stability test in vitro reveals that PSDA-2 is stable at 30–70°C and pH 3–10. Bioinformatics analysis show that PSDA-2 genome consists of 40,062 bp with a GC content of 50.21% and encodes 63 open reading frames (ORFs); no tRNA genes, lysogenic genes, drug resistance genes and virulence genes were identified in the genome. Moreover, the capacity for PSDA-2 to control *Salmonella* Typhimurium in chilled mutton was investigated. The results show that incubation of PSDA-2 at 4°C reduced recoverable *Salmonella* by 1.7 log CFU/mL and 2.1 log CFU/mL at multiplicity of infection (MOI) of 100 and 10,000 respectively, as relative to the phage-excluded control. The features of phage PSDA-2 suggest that it has the potential to be an agent to control *Salmonella*.

## Introduction

*Salmonella*, a genus of the family of *Enterobacteriaceae*, is recognized as a globally widespread foodborne pathogen. More than 2,600 *Salmonella* serotypes have been isolated up to date. Among them, nearly 99% of *Salmonella* serotypes can infect humans or animals [1, 2]. It is estimated that *Salmonella* cause 115 million human infections and 370 thousand deaths annually [3]. *Salmonella* is considered to be the highest mortality foodborne pathogen in American and the second largest zoonotic pathogen in the European Union [4].

*Salmonella* can be isolated from almost all types of food and frequently detected in meat and meat products [5]. *Salmonella* serovars are generally classified into typhoidal and nontyphoidal serovars according to whether they can cause systemic infection or localized gastroenteritis, respectively. Most outbreaks of *Salmonella* related foodborne diseases are associated with nontyphoidal *Salmonella* [6]. In nontyphoidal serovars of *Salmonella*, *Salmonella enterica* serovar Typhimurium (*S*.Typhimurium) is one of the principal serovars, which can cause acute gastroenteritis with the symptoms of diarrhea, abdominal cramps, fever, and vomiting [7, 8].

At present, chemical preservatives and physical methods are often used in food industry to prevent and control the pollution of foodborne pathogen. However, the chemical preservatives may be harmful to the body or cause quality deterioration [9]. The growing consumer demand for food products that is to avoid the use of chemical preservatives; thus, natural antimicrobial alternatives are required [10–14]. Among these natural antimicrobial agents, bacteriophage is the most promising one. Bacteriophages are widespread in the environment and do not harm beneficial bacteria because they are only specific to the host [15]. Besides, the use of phage in food does not affect its appearance, nutrition, and flavor [16]. Nowadays, the use of bacteriophage against foodborne pathogens has drawn increasing attention. Bacteriophage has proven effective in many foods, such as meat [17–20], vegetables [21, 22], milk and other liquid food [23, 24]; and some bacteriophage preparations have been put on the market and approved for use in many countries [15, 25], since FDA approved ListShield^™^ as food additives in 2006.

Mutton is one of the most popular red meats in the global market, which is estimated to be produced at around 14.326 million tons in 2015 [26]. The mutton production accounted for 23.62% of the world in 2016 and output reached 4.675 million tons in 2017 in China [27]. *Salmonella* contamination of mutton will cause great losses to enterprises and poses a great threat to public health. In this context, several novel lytic phages were isolated and characterized from the sewage sample in Hohhot, China, with PSDA-2 being one of this repertoire. The biological properties of PSDA-2 such as the one-step growth curve, lytic ability, stability of thermal and pH, and genomic information are investigated. In addition, the capacity for PSDA-2 to control *Salmonella* Typhimurium in chilled mutton was established. Overall, this study could enhance phage diversity and provide guidance for the future phage cocktail preparations as well as controlling *Salmonella* contamination of mutton.

## Materials and methods

### Bacterial strain and growth conditions

*Salmonella enterica* serovar Typhimurium (CMCC50115) was used in this study as a host. All strains (Table 1) were grown in lysogeny broth (10 g of tryptone, 5 g of yeast extract, and 5 g of NaCl per liter, pH 7.0) media at 37°C.

**Table 1:** Host range of the *Salmonella* phage PSDA-2.

Bacterial Strain	PSDA-2	Source
*Salmonella* Typhimurium	+^a^	CMCC^b^ 50115
*Salmonella* Typhimurium	+	GDMCC 1.713
*Salmonella* Typhimurium	+	GDMCC 1.1410
*Salmonella* Paratyphi-A	-	GDMCC 1.235
*Salmonella* Paratyphi-B	-	GDMCC 1.224
*Salmonella enterica* subsp. Enterica	-	ATCC 13312
*Escherichia coli* O157:H7	-	GDMCC 1.707
*Escherichia coli* DH5α	-	BNCC353719
*Klebsiella pneumoniae* subsp. Pneumoniae	-	GDMCC 1.448
*Klebsiella pneumoniae*	-	GDMCC 1.279

^a^ +, indicates cleavage; −, indicates no cleavage.

^b^CMCC, China Center for Medical Culture Collection; GDMCC, Guangdong Microbial Culture Collection Center; BNCC, BeNa Culture Collection; ATCC, American Type Culture Collection.

### Isolation and purification of phage

The double-layered agar method was used for PSDA-2 isolation, which was performed as previously described with minor modifications [28]. A sewage sample was collected in Hohhot, China, and then was filtered through 0.22 μm membrane. 10 ml of filtrate were mixed with 30 ml of exponential-phase *S*.Typhimurium (CMCC50115), and the mixture was incubated overnight at 37°C. The culture was centrifuged at 8,000 g for 10 min at 4°C, and the resulting supernatant was filtered through 0.22 μm membrane. Then 100 μL filtered supernatant with suitable dilution (diluted in SM buffer, 5.8 g of NaCl, 2.0 g of MgSO_4_ ·7H_2_O, 50 mL of Tris-HCl (pH 7.4), 5.0 mL of 2% gelatin) were mixed with 100 μL of *S*.Typhimurium, added to 5 mL of semi-solid LB (0.75% agar) after incubated about 15 min, and poured onto the surface of a prepared LB plate. Plaques appearing after an overnight incubation at 37°C were observed. The single plaque was picked aseptically and resuspended at 120 r/min in 1 mL SM buffer for 3 h, then filtered through a 0.22 μm membrane. Phage isolation by the double-layered agar method was repeated 5 times in order to purify phage. The isolates were stored at 4°C.

### Electron microscopy

Transmission electron microscopy (TEM) was applied to investigate the morphology of phage PSDA-2 [29]. Briefly, 10 μl of PEG-concentrated phages (10^11^ PFU/mL) were spotted on 400 mesh carbon-coated copper grid, and then stained with 2% phosphotungstic acid for 10 min after natural precipitation for 10~15 min. The stained PSDA-2 phage was observed using transmission electron microscopy (Hitachi HT7700) at 80 kV.

### Thermal and pH stability

Thermal stability of the phage (10^8^ PFU/mL) at different temperatures (30, 40, 50, 60, 70, 80, 90°C) was evaluated after 1 h of incubation. The effect of pH condition on phage (10^8^ PFU/mL) stability was determined at different pH levels (1~12) by incubation at 37°C for 1 h. All assays were performed in triplicate, and the phage titer was assayed by the double-layered agar method.

### Optimal multiplicity of infection

PSDA-2 and exponential-phase *S*.Typhimurium with a series of gradient multiplicity of infection (MOI) (10^−4^, 10^−3^, 10^−2^, 10^−1^ and 1) were mixed in lysogeny broth media. The mixture was incubated for 6 h at 37°C with shaking at 120 r/min. Then the culture was centrifuged at 10,000 g for 10 min, and supernatant was immediately filtered by 0.22 μm membrane. All assays were repeated in triplicate, and the phage titer was assayed by the double-layered agar method.

### One-step growth curve

*S*.Typhimurium was incubated to exponential-growth-phase and harvested by centrifugation (12,000 g, 10 min, 4°C) and resuspended in 15 mL fresh LB broth medium. The phage and its host were mixed at the optimal MOI incubated for 15 min. The mixture was centrifuged at 10,000 g for 10 min in order to remove unabsorbed phage. The precipitate was washed with 37°C LB, then the suspension was transferred to 15 mL of LB, followed by incubation at 37°C with shaking at 120 r/min. A 500 μl of phage were taken every 10 min until 120 min and immediately titrated by the double layer agar plate method. The burst size was calculated as the ratio of the final titer of liberated phages to the initial count of infected bacterial cells. All assays were repeated in three independent experiments.

### Host range

The host range of phage PSDA-2 was detected using spotting methods as previously described [30]. A total of 10 strains were used to determine the host range (Table 1), including 6 *Salmonella*, 2 *Escherichia coli* and 2 *Klebsiella*. Briefly, each strain was incubated overnight at 37°C with shaking and then 100 μL of each bacterial culture was added to 5 mL of semi-solid LB (0.75% agar) and mixed. The mixture was then poured onto the surface of a prepared LB plate. Subsequently, 10 μL of phage (10^8^ PFU/mL) with dilution in sterile SM buffer was spotted on the lawn and the plates were incubated at 37°C for 12 h. The experiment was repeated three times.

### DNA extraction, sequencing and analysis

Phage genomic DNA was extracted from concentrated PSDA-2 preparations using a Lambda phage genomic DNA Extraction Kit (Shanghai Xin Fan Biological Technology Co., Ltd). The genome sequencing was performed by Anshan Biotechnology Co., Ltd. (Tianjin, China) using Sequel Binding Kit 2.1, Sequel Sequencing Kit 2.1 and Sequel SMRT Cell 1M v2 on PacBio Sequel SMRT system. The data were processed using the SMRT link6.0 (Pacific Biosciences). Coding sequences annotation was using Prokka 1.13.3 (https://github.com/tseemann/prokka) [31]. The drug resistance gene and virulence gene were analyzed through the comprehensive antimicrobial research database (CARD, https://card.mcmaster.ca/) and the virulence factor database (VFDB, http://www.mgc.ac.cn/VFs/) respectively. The DNA polymerase sequences of the PSDA-2 and other homologous sequences obtained from the GenBank database were used to construct the phylogenetic tree. Phylogenetic analysis of the DNA polymerase was carried out using MEGA-X [32] with the neighbor connection method [33] and 1,000 bootstrap replications. Genome annotation map was constructed by the SnapGene and CG View (http://cgview.ca/). Genomic comparisons were made with Progressive MAUVE [34]. The core genes analysis was completed by CoreGenes [35].

### Effect of phage PSDA-2 on the growth of *Salmonella* in LB and chilled mutton

For the assay of phage treatment of *Salmonella* in LB, 100 μL of *S*.Typhimurium (CMCC50115) suspension (10^5^ CFU/mL) was mixed with 100 μL of phage PSDA-2 lysate (MOI = 0.0001, 0.01, 1, 100, 10000). One hundred μL of *S*.Typhimurium suspension (10^*5*^ CFU/mL) mixed with an equal volume of LB was served as a positive control whereas 100 μL of the phage lysate (10^9^ PFU/mL) mixed with an equal volume of LB was served as a negative control. The samples was incubated at 4°C for 2, 4, 8, 12, 24, 48, 72, 96,120, 144 h in 96 well plate, then, the samples were diluted to quantify *S*.Typhimurium.

For effect of phage treatment of *Salmonella* in chilled mutton, the assay was performed as previously described [36, 37] with minor modifications. Fresh chilled mutton was purchased at local market (Hohhot, Inner Mongolia, China) and cut into 1×1 cm squares under sterile conditions. Then, the meat samples were sterilized by exposure to ultraviolet (UV) light for 30 min on outside surface. The mutton was immersed in the *S*.Typhimurium (CMCC50115) suspension with a concentration of 10^4^ CFU/mL, and shaken at 120 rpm for 10 min at 4°C to uniformly distribute the host (Pre-experimental indicate that each sample can be contaminated with 10^3^ CFU/cm^2^ *S*.Typhimurium). The samples were air-dried in a vertical flow clean bench at room temperature for 15 min to allow *Salmonella* to adhere to the surface of the mutton. Subsequently, each meat was inoculated with MOI of 1,100 and 10,000 bacteriophage PSDA-2 and let stand for 15 min at room temperature to allow the phage to adsorb. As a control, SM buffer was used instead of phage suspension. The samples was incubated at 4°C for 2, 4, 8, 12, 24, 48, 72, 96,120, 144 h. Each meat sample was placed in a sterile 15 mL centrifuge tube with 5 mL of normal saline. Then, samples were mixed and diluted to quantify *S*.Typhimurium (CMCC50115). All assays were repeated in triplicate. DPS software was used to analyze the variance (ANOVA) in this experiment.

### Nucleotide sequence accession number

The complete genome sequence of *Salmonella enterica* phage PADA-2 was deposited at GenBank under accession number MW725301.

## Results

### Morphological and biological features

**Fig 1:**
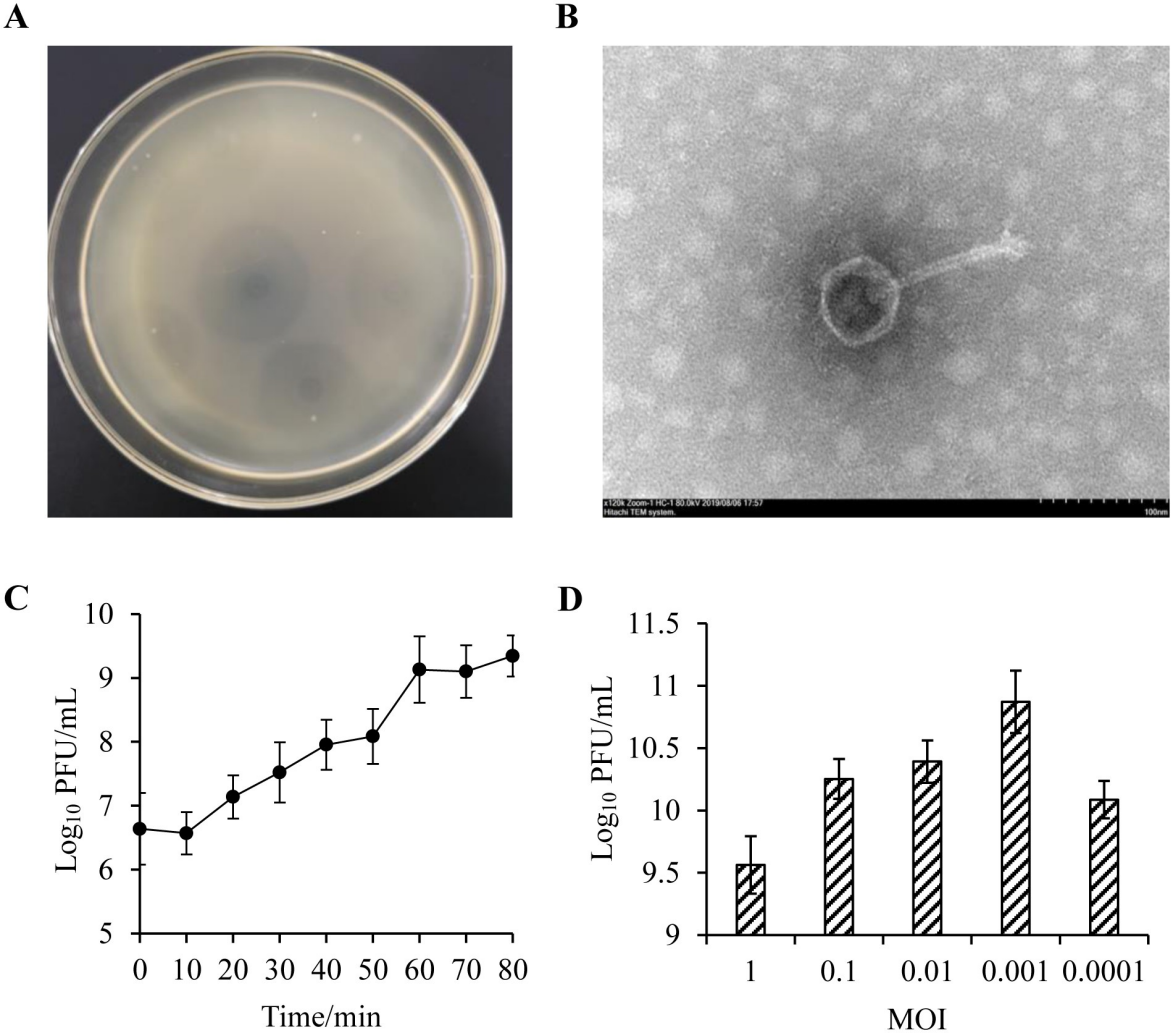
Morphological and biological features of phage PSDA-2. (A) Phage plaques formed in double-layer agar plates. (B)Transmission electron micrograph of PSDA-2. (C) One-step growth curve of PSDA-2 to host bacterial strain. (D) Titers of phage PSDA-2 under different multiplicities of infection.

A novel bacteriophage PSDA-2 was isolated and purified from sewage sample (Hohhot, Inner Mongolia, China). The PSDA-2 produced clear round plaques on the lawn of the *S*.Typhimurium (CMCC50115), and accompanied by halo circle, indicating the production of depolymerase (Fig 1A). Transmission electron microscopy revealed that PSDA-2 had an icosahedral head (isometric polyhedral head) (58 ±4 nm) and a long tail (187± 6 nm) (Fig 1B). Based on the morphology of PSDA-2, it belongs to the family *Siphoviridae*. The one-step growth curve shown that the latent period of phage PSDA-2 was 10 min and the burst period was 50 min, while burst size was 120 PFU per cell (Fig 1C). When MOI was 0.001, the titer of phage was 7.44×10^10^ PFU/mL, which was the highest compare to other MOI (Fig 1D). Therefore, the optimal MOI of PSDA-2 was 0.001.

### The stability analysis

**Fig 2:**
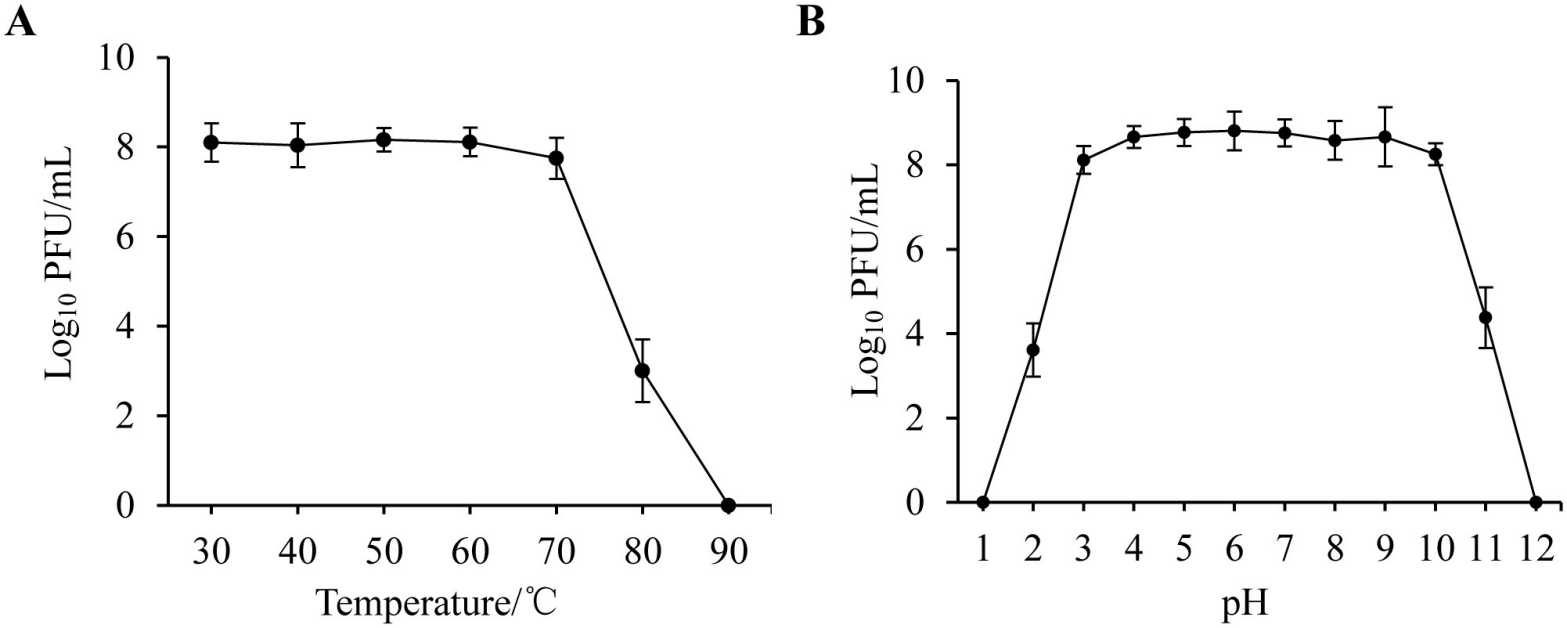
The effects of different conditions on PSDA-2 activity. (A) Temperature, (B) pH.

To test whether PSDA-2 is resistant to different environmental conditions, the survival of virions under various temperatures and pH were tested. For temperatures stability, the titer of phage PSDA-2 has no obvious variation between at 30°C and 70°C after 1 h incubation. At 80°C, phage activity decreased to one-third of that at 30°C, it was completely lost at 90°C, suggesting that phage PSDA-2 has a strong heat tolerance (Fig 2A). Meanwhile, the stability of phage PSDA-2 was investigated between pH 1 to 12. The results showed that exhibited superior active at a wide pH range that between 3 and 10, indicating that phage PSDA-2 also has an excellent tolerance to acid-base changes. There were no active phages was detected while incubation at pH 1 and 12. While at pH 2 and 11, the titer of PSDA-2 decreased 5.19 log PFU/mL and 4.43 log PFU/mL (Fig 2B).

### Host range

Host ranges of PSDA-2 was determined using a number of bacterial strains (Table 1) by spotting phage solution onto bacterial lawns. The results show that PSDA-2 is only capable of infecting three *S*.Typhimurium strains, could not infect other *Salmonella*, *Escherichia coli* and *Klebsiella pneumonia*. These results suggest the relatively strict host specificity of PSDA-2.

### Genomic features of PSDA-2

**Fig 3:**
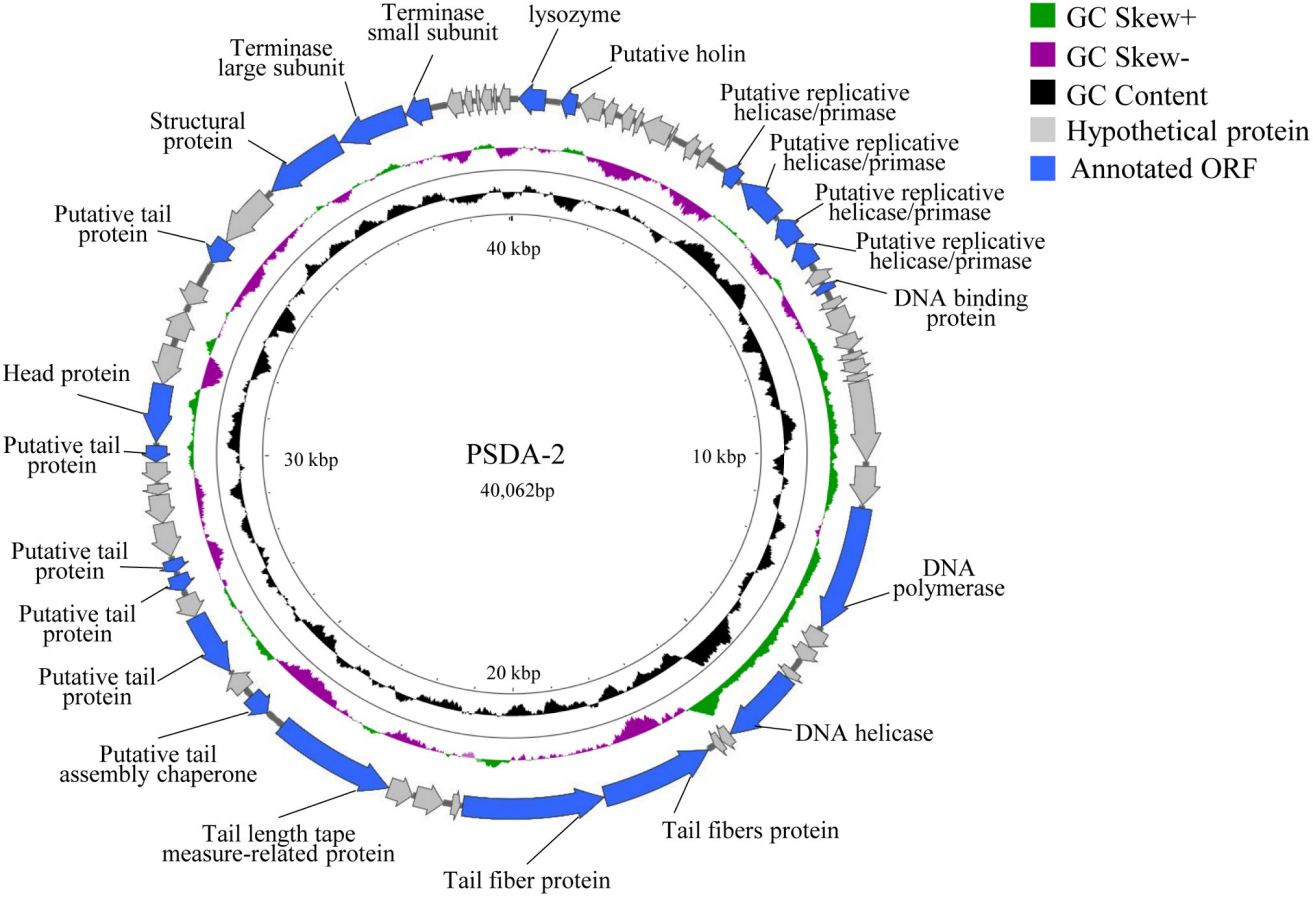
Whole genome map of phage PSDA-2.

PSDA-2 has a genome consisting of 40,062 bp with a G + C content of 50.21% (Fig 3), which is similar to the host *Salmonella*. Genomic analysis of the bacteriophage revealed 63 open reading frames (ORFs). Among them, 17 ORFs are located on the positive chain and 46 ORFs are located on the negative chain. The minimum and maximum lengths of ORFs are 117 and 2,559 bp, respectively. According to BLAST results, 24 ORFs have putative functions, and the remaining genes encode putative proteins. This may be due to the diversity of bacteriophages and insufficient database information about phage functional genes. In addition, no tRNA genes, lysogenic genes, drug resistance genes and virulence genes were identified in the genome. Similar to most bacteriophage genomes, the PSDA-2 genome is tightly packed: approximately 90% of the genome sequence encodes gene products. Based on bioinformatics predictions, the PSDA-2 genome is functionally organized into four modules, which include DNA replication/regulation, packaging, host cell lysis, and structural proteins. Seven gene products are predicted to be involved in phage DNA replication and modification, including DNA replication helicase/primerase (ORF11, ORF12, ORF13, ORF14), DNA binding protein (ORF16), DNA polymerase (ORF25), DNA helicase (ORF29). The terminase large subunit (ORF56) and the terminase small subunit (ORF57) are predicted to be related to phage packaging, which is a necessary functional protein for phage assembly. ORF1 and ORF2 are predicted to be lysozyme and holin, which involved in the host cell lysis. Genes encoding phage structural modules include tail fibrin (ORF32, ORF33), tail-length tape-related protein (ORF37), putative tail assembly protein (ORF38), putative tail protein (ORF40, ORF42, ORF43, ORF47, ORF53), head protein (ORF48), and the main capsid protein (ORF49).

### Phylogeny of phage PSDA-2

**Fig 4:**
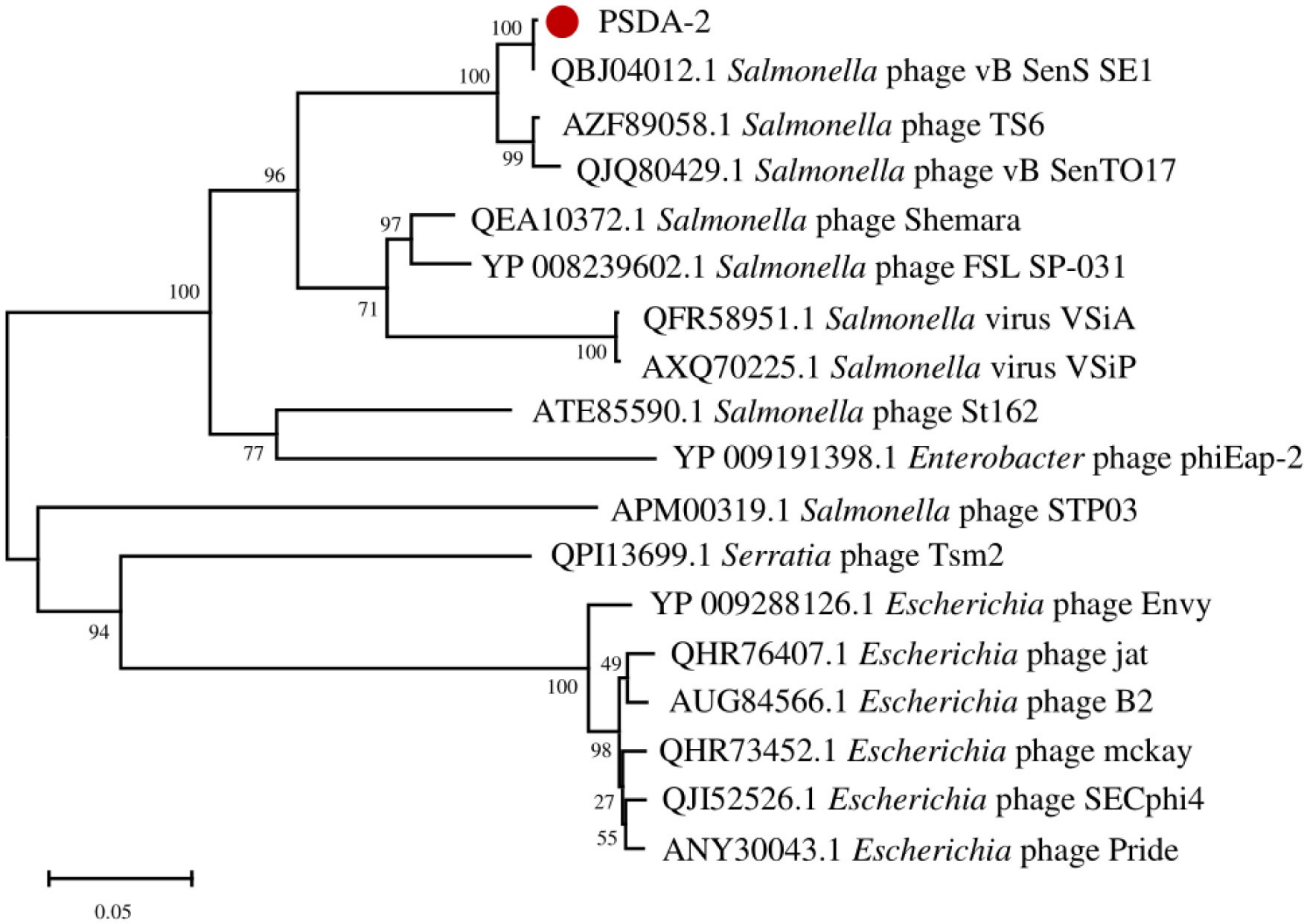
The phylogenetic tree of PSDA-2 based on DNA polymerase.

**Fig 5:**
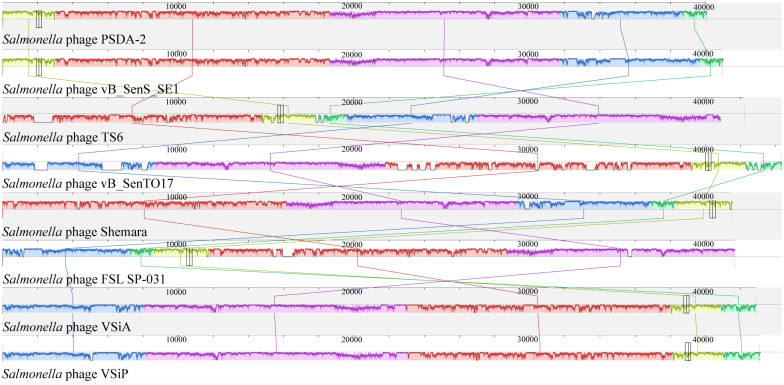
Progressive MAUVE alignment of PSDA-2 and homologous phages genome. Boxes with identical colors are homologous DNA regions shared by the chromosomes.

The phylogenetic analysis of PSDA-2 based on the DNA polymerase sequences, clearly demonstrated that PSDA-2 had sequence homology to phage vB_SenS_SE1 (Fig 4). Furthermore, the genome similarities of PSDA-2 with closely related strains (*Salmonella* phage vB_SenS_SE1, *Salmonella* phage TS6, *Salmonella* phage vB_SenTO17, *Salmonella* phage Shemara, *Salmonella* phage FSL SP-031, *Salmonella* phage VSiA and VSiP) were compared. The result showed that the gene sequence of PSDA-2 had a high degree of consistency with its relative phages, and all of these bacteriophages contain five locally collinear blocks (LCBs) (Fig 5). Meanwhile, the bioinformatic analysis using CoreGenes showed that the eight phages share 41 homologous genes in common: 65% with PSDA-2 (63 ORFs), 65% with vB_SenS_SE1 (63 ORFs), 63% with TS6 (65 ORFs), 54% with vB_SenTO17 (75 ORFs), 49% with Shemara (83 ORFs), 69% with FSL SP-031 (59 ORFs), 56% with VSiA (73 ORFs), and 56% with VSiP (73 ORFs). Phages with at least 40% homologous genes can be categorized as the same genus [38]. Therefore, PSDA-2 belong to genus *Cornellvirus*.

### Effect of phage PSDA-2 on the growth of *Salmonella* in LB and chilled mutton

**Fig 6:**
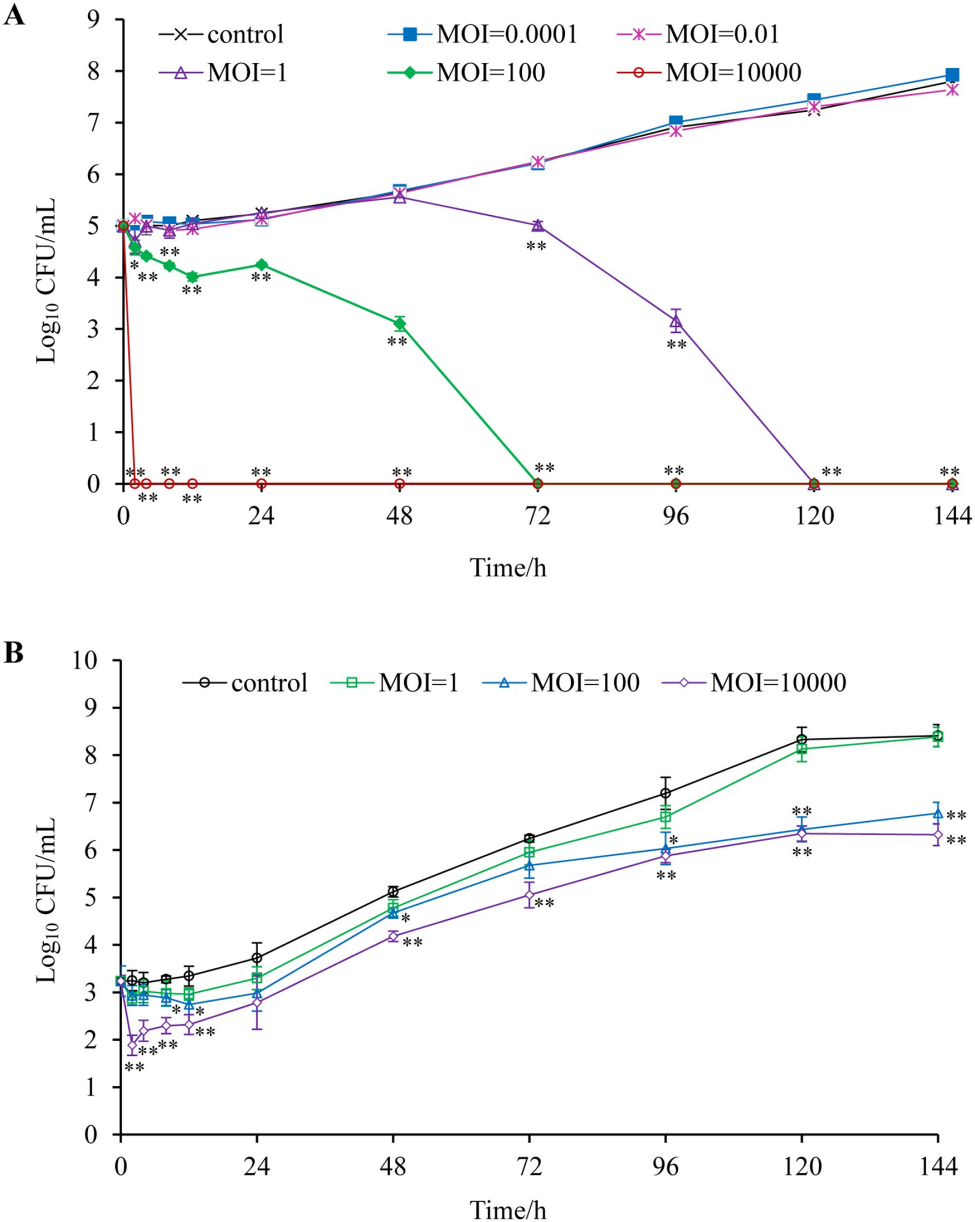
Phage PSDA-2 control of *S*.Typhimurium with different MOI. (A) In LB. (B) In chilled mutton. **, P < 0.01, *, P < 0.05, compared to the control samples at the same time point by one-way ANOVA test.

The inhibitory effect of phage on *Salmonella* increased with the increase of MOI in LB medium at 4°C. When MOI = 0.0001 and 0.01, the inhibitory effect of phage on *Salmonella* was almost invisible. When MOI = 1, 100 and 10000, *Salmonella* was completely inactivated at 120h, 72h and 2h, respectively (Fig 6A).

Under the simulated chilled mutton storage temperature (4°C), the number of *S*.Typhimurium in the control group remained basically at the initial concentration (10^3^ CFU /mL) within 12h (Fig 6B). The treatment group using PSDA-2 reduced the number of *S*.Typhimurium to varying degrees. When the MOI is 1, 100, 10,000, the number of *S*.Typhimurium decreases by about 0.4 log CFU/mL, 0.7 log CFU/mL, 1.2 log CFU/mL. With the incubation time, the number of *S*.Typhimurium in the control group gradually increased, reaching 10^8^ CFU/mL at 120 h. The number of *S*.Typhimurium in the phage-treated group with the MOI of 1 was comparable to the control group. However, the treatment group with the MOI of 100 and 10,000 observed that the number of *S*.Typhimurium was significantly lower than that of the control group. At 144 h, compared with the control group, the phage-treated group with the MOI of 100 and 10,000 reduced the number of *S*.Typhimurium by 1.7 log CFU/mL and 2.1 log CFU/mL respectively.

## Discussion

*Salmonella* is the most common foodborne pathogen in the contamination of livestock and poultry meat and their derived products. Ingestion of food contaminated by *Salmonella* may cause bacterial food poisoning. It is extremely important to prevent meat from being contaminated by *Salmonella*. In addition, *Salmonella* is very prone to antibiotic resistance, and the slow progress of new antibiotics has greatly stimulated people’s efforts to find alternatives to antibiotics. Lytic phage is considered to be the most promising alternative to antibiotics in clinical practice, and can be applied to all aspects of meat processing and production to solve the problem of foodborne bacterial contamination [39, 40]. Bacteriophages have a high degree of host specificity, so the application of bacteriophages often requires the collection of a large number of bacteriophages. Another important factor is that the phages used do not contain any genes encoding toxins or other proteins that are harmful to humans and animals. Furthermore, in the process of phages application, phages that efficiently amplify and lyse the host bacteria are preferable because they can effectively eliminate the target bacteria in a short time. Therefore, for the successful use of bacteriophages in practice, it is necessary to isolate and characterize a large number of different *Salmonella* bacteriophages [41, 42].

This study describes the isolation and identification of a *Salmonella* bacteriophage and its antibacterial application. This *Salmonella* bacteriophage was isolated from sewer sewage and named PSDA-2. Its morphology and molecular identification were carried out. Electron microscope results showed that it had an icosahedral head and a non-constricted tail. According to the International Committee on Taxonomy of viruses (ICTV), PSDA-2 can be classified into the order *Caudovirales*, the family *Siphoviridae*. The phylogenetic tree construction based on the DNA polymerase shows that PSDA-2 share the same phylogenetic branch with *Salmonella* phage vB_SenS_SE1 [43]. From the analysis of CoreGenes, PSDA-2 belongs to genus *Cornellvirus*. The plaque of PSDA-2 has a halo, which suggested the presence of a polysaccharide depolymerase. Polysaccharide depolymerase can degrade bacterial polysaccharides, including capsular polysaccharides (CPS), lipopolysaccharide (LPS), peptidoglycan, and essential components of the biofilm matrix [44]. Evidence has shown that phage-derived polysaccharide depolymerase can degrade the biofilm formed by multi-drug resistant strains and protect mice from pathogen invasion [45–48]. It is a potential antibacterial substance.

The MOI of PSDA-2 is 0.001, and the latent period is 10 min, which is shorter than *Salmonella* phage vB_SenS_SE1 (40 min), vB_SPuM_SP116 (20min) [49], SS3e (20 min) [50], and is equivalent to vB_SenTO17 (10–20 min) [51]. The exponential phase of PSDA-2 is 50 min, and the burst size is 120 pfu/cell, which is higher than vB_SenS_SE1 (19 pfu/cell), SS3e (98 pfu/cell), vB_SenTO17 (10–80 pfu/cell), and is equivalent to vB_SPuM_SP116 (118pfu/ cell). Phage PSDA-2 has a short latent period and high burst size, which can ensure the effective removal of host bacteria in a short time. In addition, the tolerance and host spectrum of phages are also the most important considerations for their potential application. Compared with vB_SenS_SE1, the potency of PSDA-2 begins to decrease when the temperature is greater than 70 degrees, while vB_SenS_SE1 begins to lose its potency when the temperature is greater than 50 degrees [43]. The titer of PSDA-2 is almost unchanged between pH 3–10, which makes it suitable for a wide range of applications. PSDA-2 can only lyse *S*.Typhimurium within the range of tested strains, which is similar to the evolutionary related phages vB_SenS_SE1 and vB_SenTO17 [51]. But limited by the small number of bacterial strains used for host range, it can only be concluded that PSDA-2 has the relatively strict host specificity. The high specificity of bacteriophages is its greatest advantage, and it is also one of the obstacles that limit its practical application. Precise targeting is an important consideration for any prevention and treatment method. The high specificity of bacteriophages is the guarantee of accurate targeting. However, when many different species and intraspecies variants need treatment, just like the treatment of typical bacterial infection, the system needs flexibility to target a large part of these variants. Therefore, appropriately broadening and changing the host range of phage is an important step to realize the application. Although it has been reported that the host spectrum of phages is changed by engineering methods [52–54], but it is limited to several phages that clarify the mechanism of receptor binding protein adsorption on the host, which is still a long way from practical application. At present, the main method to broaden the host range of phages is to mix phages with different host ranges, which is called a phage cocktail. The excellent properties of phage PSDA-2 can make it have the potential to formulate cocktail preparations.

But not all phages can be used to control pathogens. For safety reasons, phages with antibiotic resistance, lysogenic and pathogenic genes should not be used as biological control agents in the food industry. Studies have shown that bacteriophages containing genes encoding some virulence factors synthesize virulence factors after entering the host bacterial cells, causing harm to human body. Bacteriophages may also be the medium for the horizontal transmission of drug-resistant genes among drug-resistant bacteria [55, 56]. Therefore, genetic analysis can further identify whether the new phage isolates have potential application value. The genome length of PSDA-2 is 40062bp, which is the smallest among *Salmonella* phage with similar genetic relationship, including phage vB_SenS_SE1 (40987 bp, MK479295.1), TS6 (41515 bp, MK214385.1), vB_SenTO17 (41658 bp, MT012729.1), Shemara (44342 bp, MN070121.2), FSL SP-031 (42215 bp, NC_021775.1), VSiA (43110 bp, MN393079.1) and VsiP (42865 bp, MH424444.1). The GC content is 50.21%, which was similar to that of host *Salmonella* (about 52.2%). PSDA-2 contains 63 ORFs, of which 24 have annotated functions, and the rest are hypothetical proteins. Among them, ORF1 and ORF2 are predicted to be lysozyme and holin. These two proteins play an important role in the process of phage invasion and lysis of the host. They are also antibacterial substances with broad application prospects [57]. Lysozyme, also known as N-acetylmuramidases, is a type of endolysin in phage [58]. The lysozyme of PSDA-2 is almost 100% similar to T4 phage lysozyme, and T4 phage lysozyme completed its structural analysis as early as 1987 [59]. Holin is a small polar transmembrane protein synthesized in the late stage of phage infection. It can cause non-specific damage to the bacterial cytoplasmic membrane and form a stable transmembrane pore. Endolysin can reach the peptidoglycan layer of cell wall through this pore and play the role of bacteriolysis [60, 61]. PSDA-2 hole protein has 98.92% and 97.85% similarity with the hypothetical protein derived from phage St161 and St162, respectively, and 74.19% similarity with the hypothetical protein gp50 from phage Ent1 [62]. No tRNA coding gene was found in PSDA-2 genome, indicating that it relies on the host tRNA to complete its protein synthesis. In addition, no lysogenic genes were found in the PSDA-2 genome, and no drug resistance gene and virulence gene were found through the search of the comprehensive antimicrobial research database (CARD) and the virulence factor database (VFDB). Genomic analysis showed that PSDA-2 is a safe lytic phage.

Some studies have shown that the properties of food matrix will affect the inhibitory effect of bacteriophages on the host [63], so it is necessary to evaluate its antibacterial effect in various food matrices before phage application. The purpose of this study was to isolate bacteriophages that can be used to prevent and control *Salmonella* contamination in chilled mutton, so we further evaluated the inhibitory effect of phage PSDA-2 on *S*.Typhimurium under specific conditions. The addition of low titer PSDA-2 (MOI = 0.0001 and 0.01) had no inhibitory effect on *S*.Typhimurium in LB medium at 4°C. With the increase of titer (MOI = 1, 100, 10000), the inhibitory effect of phage on *S*.Typhimurium was enhanced and the time of to eliminate *S*.Typhimurium was shortened. The results showed that phages with different titers inhibited *Salmonella* contamination in chilled mutton within 120 hours after phage addition, and the decrease of *S*.Typhimurium was positively correlated with MOI. When phages were added for 144 hours, the number of *S*.Typhimurium in the experimental group with MOI of 1 was almost the same as that of the control group, and the experimental group with MOI of 100 and 10 000 had different degrees of inhibition on *S*.Typhimurium. The antibacterial effect of adding high titer of phages was significantly better than that of adding low titer. Similar studies have shown that phages have a good effect on inhibiting *Salmonella* in other food substrates. Respectively, broad spectrum phages LPSE1 and LPST10 can inhibit the growth of *S*.Enteritidis (ATCC13076) and *S*.Typhimurium (ATCC14028) to varying degrees in milk, usage and lettuce [37, 64]. Phage D1-2 can effectively inhibit the growth of multidrug resistant *Salmonella* at 4°C and 25°C in egg white and egg yolk [65]. However, the food matrix may affect the inhibitory effect of phages by influencing the emergence of phage resistance. In foods with less complex microbial communities, the resistance develops faster [63]. The titer of phages is undoubtedly an important factor affecting the antibacterial effect of phage [63]. Some studies have shown that the use of high titer of bacteriophages can usually achieve a high reduction rate of pathogen [36, 66]. In comparison, regardless of the phage specificity, the antibacterial effect of a single phage is lower than that of a phage cocktail preparation [30, 67], which is due to the fact that the use of a single phage is more likely to lead to the production of resistant host bacteria [68]. In summary, this study laid a foundation for the further preparation of phage cocktail.

## Supporting information

S1 File (RAR)

S1 Fig Adsorption assay of PSDA-2 to host strain *S*.Typhimurium (CMCC50115). (TIF)

S1 Table The number of homologous genes in the eight phages. (DOCX)
