# Supplementary figures and images for: Isolation, characterization and application of bacteriophage PSDA-2 against Salmonella Typhimurium in chilled mutton
Source: PLoS One. 2022 Jan 24;17(1):e0262946. doi: 10.1371/journal.pone.0262946 (PMC8786174; doi:10.1371/journal.pone.0262946)

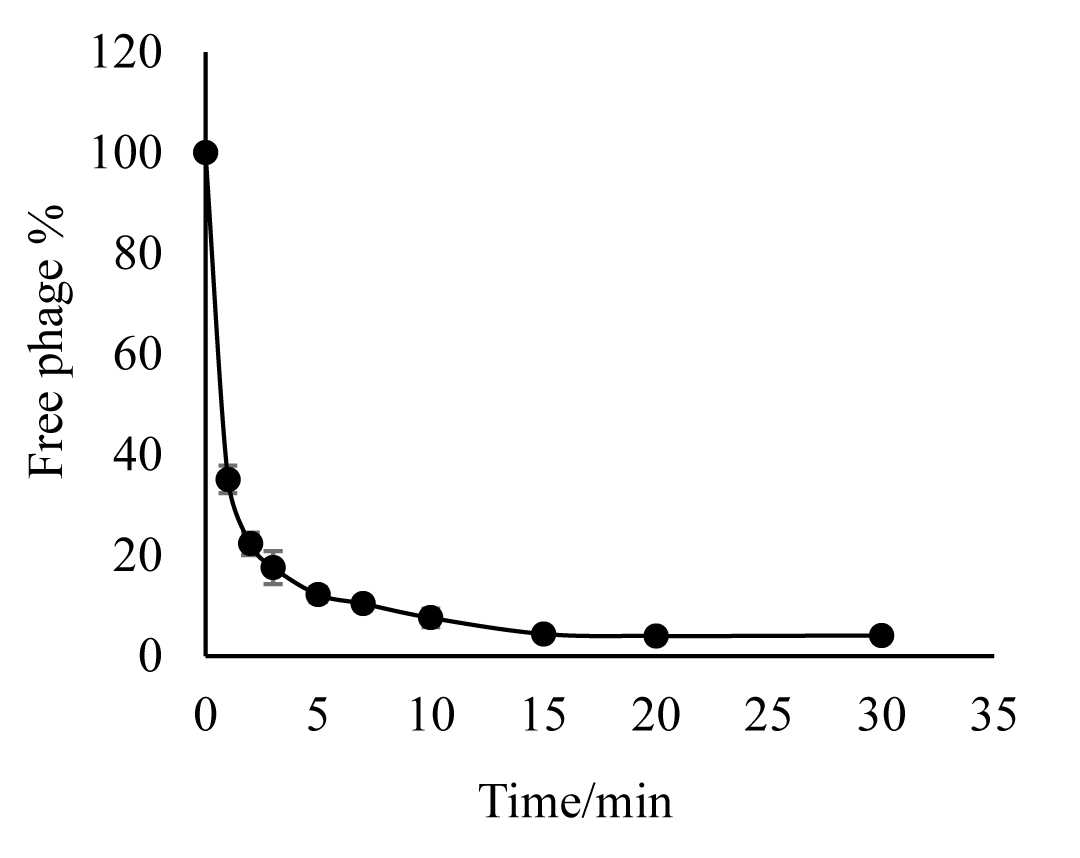

Supplement: S1 Fig — (TIF) [file pone.0262946.s002.tif]
