# Supplementary material for: Isolation, characterization and application of bacteriophage PSDA-2 against Salmonella Typhimurium in chilled mutton
Source: PLoS One. 2022 Jan 24;17(1):e0262946. doi: 10.1371/journal.pone.0262946 (PMC8786174; doi:10.1371/journal.pone.0262946)
Supplement: S1 Table — (DOCX) [file pone.0262946.s003.docx]

| ***Salmonella* phage PSDA-2** [***MW725301.1***](https://www.ncbi.nlm.nih.gov/nuccore/MW725301.1&doptcmdl=GenBank) | ***Salmonella* phage vB_SenS_SE1** [***MK479295.1***](https://www.ncbi.nlm.nih.gov/nuccore/MK479295.1&doptcmdl=GenBank) | ***Salmonella* phage TS6** [***MK214385.1***](https://www.ncbi.nlm.nih.gov/nuccore/MK214385.1&doptcmdl=GenBank) | ***Salmonella* phage vB_SenTO17** [***MT012729.1***](https://www.ncbi.nlm.nih.gov/nuccore/MT012729.1&doptcmdl=GenBank) | ***Salmonella* phage Shemara** [***MN070121.2***](https://www.ncbi.nlm.nih.gov/nuccore/MN070121.2&doptcmdl=GenBank) | ***Salmonella* phage FSL SP-031** [***NC_021775.1***](https://www.ncbi.nlm.nih.gov/nuccore/NC_021775.1&doptcmdl=GenBank) | ***Salmonella* virus VSiA** [***MN393079.1***](https://www.ncbi.nlm.nih.gov/nuccore/MN393079.1&doptcmdl=GenBank) | ***Salmonella* virus VSiP** [***MH424444.1***](https://www.ncbi.nlm.nih.gov/nuccore/MH424444.1&doptcmdl=GenBank) |
| --- | --- | --- | --- | --- | --- | --- | --- |
| PI:[*QVW27635.1*](https://www.ncbi.nlm.nih.gov/nuccore/QVW27635.1&doptcmdl=GenBank) Product:lysozyme | PI:[*QBJ03990.1*](https://www.ncbi.nlm.nih.gov/nuccore/QBJ03990.1&doptcmdl=GenBank) Product:lysozyme | PI:[*AZF89100.1*](https://www.ncbi.nlm.nih.gov/nuccore/AZF89100.1&doptcmdl=GenBank) Product:lysin | PI:[*QJQ80402.1*](https://www.ncbi.nlm.nih.gov/nuccore/QJQ80402.1&doptcmdl=GenBank) Product:lysin | PI:[*QEA10400.1*](https://www.ncbi.nlm.nih.gov/nuccore/QEA10400.1&doptcmdl=GenBank) Product:endolysin | PI:[*YP_008239621.1*](https://www.ncbi.nlm.nih.gov/nuccore/YP_008239621.1&doptcmdl=GenBank) Product:lysozyme | PI:[*QFR58975.1*](https://www.ncbi.nlm.nih.gov/nuccore/QFR58975.1&doptcmdl=GenBank) Product:lysin | PI:[*AXQ70248.1*](https://www.ncbi.nlm.nih.gov/nuccore/AXQ70248.1&doptcmdl=GenBank) Product:lysin |
| PI:[*QVW27636.1*](https://www.ncbi.nlm.nih.gov/nuccore/QVW27636.1&doptcmdl=GenBank) Product:putative holin | PI:[*QBJ03991.1*](https://www.ncbi.nlm.nih.gov/nuccore/QBJ03991.1&doptcmdl=GenBank) Product:putative holin | PI:[*AZF89102.1*](https://www.ncbi.nlm.nih.gov/nuccore/AZF89102.1&doptcmdl=GenBank) Product:hypothetical protein | PI:[*QJQ80404.1*](https://www.ncbi.nlm.nih.gov/nuccore/QJQ80404.1&doptcmdl=GenBank) Product:putative holin | PI:[*QEA10398.1*](https://www.ncbi.nlm.nih.gov/nuccore/QEA10398.1&doptcmdl=GenBank) Product:putative holin/antiholin | PI:[*YP_008239619.1*](https://www.ncbi.nlm.nih.gov/nuccore/YP_008239619.1&doptcmdl=GenBank) Product:hypothetical protein | PI:[*QFR58973.1*](https://www.ncbi.nlm.nih.gov/nuccore/QFR58973.1&doptcmdl=GenBank) Product:hypothetical protein | PI:[*AXQ70246.1*](https://www.ncbi.nlm.nih.gov/nuccore/AXQ70246.1&doptcmdl=GenBank) Product:hypothetical protein |
| PI:[*QVW27637.1*](https://www.ncbi.nlm.nih.gov/nuccore/QVW27637.1&doptcmdl=GenBank) Product:hypothetical protein | PI:[*QBJ03992.1*](https://www.ncbi.nlm.nih.gov/nuccore/QBJ03992.1&doptcmdl=GenBank) Product:hypothetical protein | PI:[*AZF89103.1*](https://www.ncbi.nlm.nih.gov/nuccore/AZF89103.1&doptcmdl=GenBank) Product:hypothetical protein | PI:[*QJQ80405.1*](https://www.ncbi.nlm.nih.gov/nuccore/QJQ80405.1&doptcmdl=GenBank) Product:hypothetical protein | PI:[*QEA10397.1*](https://www.ncbi.nlm.nih.gov/nuccore/QEA10397.1&doptcmdl=GenBank) Product:hypothetical protein | PI:[*YP_008239618.1*](https://www.ncbi.nlm.nih.gov/nuccore/YP_008239618.1&doptcmdl=GenBank) Product:hypothetical protein | PI:[*QFR58972.1*](https://www.ncbi.nlm.nih.gov/nuccore/QFR58972.1&doptcmdl=GenBank) Product:hypothetical protein | PI:[*AXQ70245.1*](https://www.ncbi.nlm.nih.gov/nuccore/AXQ70245.1&doptcmdl=GenBank) Product:hypothetical protein |
| PI:[*QVW27640.1*](https://www.ncbi.nlm.nih.gov/nuccore/QVW27640.1&doptcmdl=GenBank) Product:hypothetical protein | PI:[*QBJ03995.1*](https://www.ncbi.nlm.nih.gov/nuccore/QBJ03995.1&doptcmdl=GenBank) Product:hypothetical protein | PI:[*AZF89106.1*](https://www.ncbi.nlm.nih.gov/nuccore/AZF89106.1&doptcmdl=GenBank) Product:hypothetical protein | PI:[*QJQ80408.1*](https://www.ncbi.nlm.nih.gov/nuccore/QJQ80408.1&doptcmdl=GenBank) Product:hypothetical protein | PI:[*QEA10394.1*](https://www.ncbi.nlm.nih.gov/nuccore/QEA10394.1&doptcmdl=GenBank) Product:hypothetical protein | PI:[*YP_008239616.1*](https://www.ncbi.nlm.nih.gov/nuccore/YP_008239616.1&doptcmdl=GenBank) Product:hypothetical protein | PI:[*QFR58969.1*](https://www.ncbi.nlm.nih.gov/nuccore/QFR58969.1&doptcmdl=GenBank) Product:hypothetical protein | PI:[*AXQ70242.1*](https://www.ncbi.nlm.nih.gov/nuccore/AXQ70242.1&doptcmdl=GenBank) Product:hypothetical protein |
| PI:[*QVW27641.1*](https://www.ncbi.nlm.nih.gov/nuccore/QVW27641.1&doptcmdl=GenBank) Product:hypothetical protein | PI:[*QBJ03996.1*](https://www.ncbi.nlm.nih.gov/nuccore/QBJ03996.1&doptcmdl=GenBank) Product:hypothetical protein | PI:[*AZF89107.1*](https://www.ncbi.nlm.nih.gov/nuccore/AZF89107.1&doptcmdl=GenBank) Product:hypothetical protein | PI:[*QJQ80409.1*](https://www.ncbi.nlm.nih.gov/nuccore/QJQ80409.1&doptcmdl=GenBank) Product:hypothetical protein | PI:[*QEA10393.1*](https://www.ncbi.nlm.nih.gov/nuccore/QEA10393.1&doptcmdl=GenBank) Product:hypothetical protein | PI:[*YP_008239615.1*](https://www.ncbi.nlm.nih.gov/nuccore/YP_008239615.1&doptcmdl=GenBank) Product:hypothetical protein | PI:[*QFR58968.1*](https://www.ncbi.nlm.nih.gov/nuccore/QFR58968.1&doptcmdl=GenBank) Product:hypothetical protein | PI:[*AXQ70241.1*](https://www.ncbi.nlm.nih.gov/nuccore/AXQ70241.1&doptcmdl=GenBank) Product:hypothetical protein |
| PI:[*QVW27646.1*](https://www.ncbi.nlm.nih.gov/nuccore/QVW27646.1&doptcmdl=GenBank) Product:putative replicative helicase/primase | PI:[*QBJ04001.1*](https://www.ncbi.nlm.nih.gov/nuccore/QBJ04001.1&doptcmdl=GenBank) Product:putative replicative helicase/primase | PI:[*AZF89047.1*](https://www.ncbi.nlm.nih.gov/nuccore/AZF89047.1&doptcmdl=GenBank) Product:replicative DNA helicase | PI:[*QJQ80417.1*](https://www.ncbi.nlm.nih.gov/nuccore/QJQ80417.1&doptcmdl=GenBank) Product:putative replicative helicase/primease | PI:[*QEA10386.1*](https://www.ncbi.nlm.nih.gov/nuccore/QEA10386.1&doptcmdl=GenBank) Product:P-loop NTPase domain-containing protein | PI:[*YP_008239613.1*](https://www.ncbi.nlm.nih.gov/nuccore/YP_008239613.1&doptcmdl=GenBank) Product:putative phage replicative helicase/primease | PI:[*QFR58963.1*](https://www.ncbi.nlm.nih.gov/nuccore/QFR58963.1&doptcmdl=GenBank) Product:DNA helicase/primase | PI:[*AXQ70237.1*](https://www.ncbi.nlm.nih.gov/nuccore/AXQ70237.1&doptcmdl=GenBank) Product:DNA helicase/primase |
| PI:[*QVW27650.1*](https://www.ncbi.nlm.nih.gov/nuccore/QVW27650.1&doptcmdl=GenBank) Product:DNA binding protein | PI:[*QBJ04003.1*](https://www.ncbi.nlm.nih.gov/nuccore/QBJ04003.1&doptcmdl=GenBank) Product:DNA binding protein | PI:[*AZF89049.1*](https://www.ncbi.nlm.nih.gov/nuccore/AZF89049.1&doptcmdl=GenBank) Product:hypothetical protein | PI:[*QJQ80419.1*](https://www.ncbi.nlm.nih.gov/nuccore/QJQ80419.1&doptcmdl=GenBank) Product:DNA-binding protein | PI:[*QEA10383.1*](https://www.ncbi.nlm.nih.gov/nuccore/QEA10383.1&doptcmdl=GenBank) Product:transcriptional regulator | PI:[*YP_008239612.1*](https://www.ncbi.nlm.nih.gov/nuccore/YP_008239612.1&doptcmdl=GenBank) Product:hypothetical protein | PI:[*QFR58962.1*](https://www.ncbi.nlm.nih.gov/nuccore/QFR58962.1&doptcmdl=GenBank) Product:hypothetical protein | PI:[*AXQ70236.1*](https://www.ncbi.nlm.nih.gov/nuccore/AXQ70236.1&doptcmdl=GenBank) Product:hypothetical protein |
| PI:[*QVW27652.1*](https://www.ncbi.nlm.nih.gov/nuccore/QVW27652.1&doptcmdl=GenBank) Product:hypothetical protein | PI:[*QBJ04005.1*](https://www.ncbi.nlm.nih.gov/nuccore/QBJ04005.1&doptcmdl=GenBank) Product:hypothetical protein | PI:[*AZF89051.1*](https://www.ncbi.nlm.nih.gov/nuccore/AZF89051.1&doptcmdl=GenBank) Product:hypothetical protein | PI:[*QJQ80422.1*](https://www.ncbi.nlm.nih.gov/nuccore/QJQ80422.1&doptcmdl=GenBank) Product:hypothetical protein | PI:[*QEA10381.1*](https://www.ncbi.nlm.nih.gov/nuccore/QEA10381.1&doptcmdl=GenBank) Product:hypothetical protein | PI:[*YP_008239611.1*](https://www.ncbi.nlm.nih.gov/nuccore/YP_008239611.1&doptcmdl=GenBank) Product:hypothetical protein | PI:[*QFR58960.1*](https://www.ncbi.nlm.nih.gov/nuccore/QFR58960.1&doptcmdl=GenBank) Product:hypothetical protein | PI:[*AXQ70234.1*](https://www.ncbi.nlm.nih.gov/nuccore/AXQ70234.1&doptcmdl=GenBank) Product:hypothetical protein |
| PI:[*QVW27655.1*](https://www.ncbi.nlm.nih.gov/nuccore/QVW27655.1&doptcmdl=GenBank) Product:hypothetical protein | PI:[*QBJ04008.1*](https://www.ncbi.nlm.nih.gov/nuccore/QBJ04008.1&doptcmdl=GenBank) Product:hypothetical protein | PI:[*AZF89054.1*](https://www.ncbi.nlm.nih.gov/nuccore/AZF89054.1&doptcmdl=GenBank) Product:hypothetical protein | PI:[*QJQ80425.1*](https://www.ncbi.nlm.nih.gov/nuccore/QJQ80425.1&doptcmdl=GenBank) Product:hypothetical protein | PI:[*QEA10378.1*](https://www.ncbi.nlm.nih.gov/nuccore/QEA10378.1&doptcmdl=GenBank) Product:hypothetical protein | PI:[*YP_008239608.1*](https://www.ncbi.nlm.nih.gov/nuccore/YP_008239608.1&doptcmdl=GenBank) Product:hypothetical protein | PI:[*QFR58957.1*](https://www.ncbi.nlm.nih.gov/nuccore/QFR58957.1&doptcmdl=GenBank) Product:hypothetical protein | PI:[*AXQ70231.1*](https://www.ncbi.nlm.nih.gov/nuccore/AXQ70231.1&doptcmdl=GenBank) Product:hypothetical protein |
| PI:[*QVW27657.1*](https://www.ncbi.nlm.nih.gov/nuccore/QVW27657.1&doptcmdl=GenBank) Product:hypothetical protein | PI:[*QBJ04010.1*](https://www.ncbi.nlm.nih.gov/nuccore/QBJ04010.1&doptcmdl=GenBank) Product:hypothetical protein | PI:[*AZF89056.1*](https://www.ncbi.nlm.nih.gov/nuccore/AZF89056.1&doptcmdl=GenBank) Product:hypothetical protein | PI:[*QJQ80427.1*](https://www.ncbi.nlm.nih.gov/nuccore/QJQ80427.1&doptcmdl=GenBank) Product:hypothetical protein | PI:[*QEA10376.1*](https://www.ncbi.nlm.nih.gov/nuccore/QEA10376.1&doptcmdl=GenBank) Product:hypothetical protein | PI:[*YP_008239606.1*](https://www.ncbi.nlm.nih.gov/nuccore/YP_008239606.1&doptcmdl=GenBank) Product:hypothetical protein | PI:[*QFR58955.1*](https://www.ncbi.nlm.nih.gov/nuccore/QFR58955.1&doptcmdl=GenBank) Product:DUF2800 domain-containing protein | PI:[*AXQ70229.1*](https://www.ncbi.nlm.nih.gov/nuccore/AXQ70229.1&doptcmdl=GenBank) Product:hypothetical protein |
| PI:[*QVW27658.1*](https://www.ncbi.nlm.nih.gov/nuccore/QVW27658.1&doptcmdl=GenBank) Product:hypothetical protein | PI:[*QBJ04011.1*](https://www.ncbi.nlm.nih.gov/nuccore/QBJ04011.1&doptcmdl=GenBank) Product:hypothetical protein | PI:[*AZF89057.1*](https://www.ncbi.nlm.nih.gov/nuccore/AZF89057.1&doptcmdl=GenBank) Product:hypothetical protein | PI:[*QJQ80428.1*](https://www.ncbi.nlm.nih.gov/nuccore/QJQ80428.1&doptcmdl=GenBank) Product:hypothetical protein | PI:[*QEA10374.1*](https://www.ncbi.nlm.nih.gov/nuccore/QEA10374.1&doptcmdl=GenBank) Product:nucleic acid-binding domain-containing protein | PI:[*YP_008239604.1*](https://www.ncbi.nlm.nih.gov/nuccore/YP_008239604.1&doptcmdl=GenBank) Product:hypothetical protein | PI:[*QFR58953.1*](https://www.ncbi.nlm.nih.gov/nuccore/QFR58953.1&doptcmdl=GenBank) Product:DUF2815 domain-containing protein | PI:[*AXQ70227.1*](https://www.ncbi.nlm.nih.gov/nuccore/AXQ70227.1&doptcmdl=GenBank) Product:hypothetical protein |
| PI:[*QVW27659.1*](https://www.ncbi.nlm.nih.gov/nuccore/QVW27659.1&doptcmdl=GenBank) Product:DNA polymerase I | PI:[*QBJ04012.1*](https://www.ncbi.nlm.nih.gov/nuccore/QBJ04012.1&doptcmdl=GenBank) Product:DNA polymerase I | PI:[*AZF89058.1*](https://www.ncbi.nlm.nih.gov/nuccore/AZF89058.1&doptcmdl=GenBank) Product:DNA polymerase I | PI:[*QJQ80429.1*](https://www.ncbi.nlm.nih.gov/nuccore/QJQ80429.1&doptcmdl=GenBank) Product:DNA polymerase I | PI:[*QEA10372.1*](https://www.ncbi.nlm.nih.gov/nuccore/QEA10372.1&doptcmdl=GenBank) Product:DNA polymerase | PI:[*YP_008239602.1*](https://www.ncbi.nlm.nih.gov/nuccore/YP_008239602.1&doptcmdl=GenBank) Product:DNA polymerase I | PI:[*QFR58951.1*](https://www.ncbi.nlm.nih.gov/nuccore/QFR58951.1&doptcmdl=GenBank) Product:DNA polymerase | PI:[*AXQ70225.1*](https://www.ncbi.nlm.nih.gov/nuccore/AXQ70225.1&doptcmdl=GenBank) Product:DNA polymerase |
| PI:[*QVW27661.1*](https://www.ncbi.nlm.nih.gov/nuccore/QVW27661.1&doptcmdl=GenBank) Product:putative endonuclease | PI:[*QBJ04014.1*](https://www.ncbi.nlm.nih.gov/nuccore/QBJ04014.1&doptcmdl=GenBank) Product:putative endonuclease | PI:[*AZF89061.1*](https://www.ncbi.nlm.nih.gov/nuccore/AZF89061.1&doptcmdl=GenBank) Product:hypothetical protein | PI:[*QJQ80432.1*](https://www.ncbi.nlm.nih.gov/nuccore/QJQ80432.1&doptcmdl=GenBank) Product:hypothetical protein | PI:[*QEA10368.1*](https://www.ncbi.nlm.nih.gov/nuccore/QEA10368.1&doptcmdl=GenBank) Product:nuclease | PI:[*YP_008239600.1*](https://www.ncbi.nlm.nih.gov/nuccore/YP_008239600.1&doptcmdl=GenBank) Product:hypothetical protein | PI:[*QFR58949.1*](https://www.ncbi.nlm.nih.gov/nuccore/QFR58949.1&doptcmdl=GenBank) Product:hypothetical protein | PI:[*AXQ70223.1*](https://www.ncbi.nlm.nih.gov/nuccore/AXQ70223.1&doptcmdl=GenBank) Product:hypothetical protein |
| PI:[*QVW27663.1*](https://www.ncbi.nlm.nih.gov/nuccore/QVW27663.1&doptcmdl=GenBank) Product:DNA helicase | PI:[*QBJ04016.1*](https://www.ncbi.nlm.nih.gov/nuccore/QBJ04016.1&doptcmdl=GenBank) Product:DNA helicase | PI:[*AZF89063.1*](https://www.ncbi.nlm.nih.gov/nuccore/AZF89063.1&doptcmdl=GenBank) Product:DUF2740 domain-dontaining protein | PI:[*QJQ80436.1*](https://www.ncbi.nlm.nih.gov/nuccore/QJQ80436.1&doptcmdl=GenBank) Product:DNA helicase | PI:[*QEA10364.1*](https://www.ncbi.nlm.nih.gov/nuccore/QEA10364.1&doptcmdl=GenBank) Product:helicase | PI:[*YP_008239599.1*](https://www.ncbi.nlm.nih.gov/nuccore/YP_008239599.1&doptcmdl=GenBank) Product:helicase | PI:[*QFR58945.1*](https://www.ncbi.nlm.nih.gov/nuccore/QFR58945.1&doptcmdl=GenBank) Product:DNA helicase | PI:[*AXQ70219.1*](https://www.ncbi.nlm.nih.gov/nuccore/AXQ70219.1&doptcmdl=GenBank) Product:DNA helicase |
| PI:[*QVW27665.1*](https://www.ncbi.nlm.nih.gov/nuccore/QVW27665.1&doptcmdl=GenBank) Product:hypothetical protein | PI:[*QBJ04018.1*](https://www.ncbi.nlm.nih.gov/nuccore/QBJ04018.1&doptcmdl=GenBank) Product:hypothetical protein | PI:[*AZF89065.1*](https://www.ncbi.nlm.nih.gov/nuccore/AZF89065.1&doptcmdl=GenBank) Product:hypothetical protein | PI:[*QJQ80438.1*](https://www.ncbi.nlm.nih.gov/nuccore/QJQ80438.1&doptcmdl=GenBank) Product:hypothetical protein | PI:[*QEA10362.1*](https://www.ncbi.nlm.nih.gov/nuccore/QEA10362.1&doptcmdl=GenBank) Product:hypothetical protein | PI:[*YP_008239598.1*](https://www.ncbi.nlm.nih.gov/nuccore/YP_008239598.1&doptcmdl=GenBank) Product:hypothetical protein | PI:[*QFR58943.1*](https://www.ncbi.nlm.nih.gov/nuccore/QFR58943.1&doptcmdl=GenBank) Product:hypothetical protein | PI:[*AXQ70217.1*](https://www.ncbi.nlm.nih.gov/nuccore/AXQ70217.1&doptcmdl=GenBank) Product:hypothetical protein |
| PI:[*QVW27667.1*](https://www.ncbi.nlm.nih.gov/nuccore/QVW27667.1&doptcmdl=GenBank) Product:tail fiber protein | PI:[*QBJ04020.1*](https://www.ncbi.nlm.nih.gov/nuccore/QBJ04020.1&doptcmdl=GenBank) Product:tail fiber protein | PI:[*AZF89067.1*](https://www.ncbi.nlm.nih.gov/nuccore/AZF89067.1&doptcmdl=GenBank) Product:hypothetical protein | PI:[*QJQ80441.1*](https://www.ncbi.nlm.nih.gov/nuccore/QJQ80441.1&doptcmdl=GenBank) Product:tail protein | PI:[*QEA10359.1*](https://www.ncbi.nlm.nih.gov/nuccore/QEA10359.1&doptcmdl=GenBank) Product:tail fiber protein | PI:[*YP_008239655.1*](https://www.ncbi.nlm.nih.gov/nuccore/YP_008239655.1&doptcmdl=GenBank) Product:tail fiber protein | PI:[*QFR58941.1*](https://www.ncbi.nlm.nih.gov/nuccore/QFR58941.1&doptcmdl=GenBank) Product:tail fiber protein | PI:[*AXQ70215.1*](https://www.ncbi.nlm.nih.gov/nuccore/AXQ70215.1&doptcmdl=GenBank) Product:tail fiber protein |
| PI:[*QVW27668.1*](https://www.ncbi.nlm.nih.gov/nuccore/QVW27668.1&doptcmdl=GenBank) Product:hypothetical protein | PI:[*QBJ04021.1*](https://www.ncbi.nlm.nih.gov/nuccore/QBJ04021.1&doptcmdl=GenBank) Product:hypothetical protein | PI:[*AZF89068.1*](https://www.ncbi.nlm.nih.gov/nuccore/AZF89068.1&doptcmdl=GenBank) Product:hypothetical protein | PI:[*QJQ80442.1*](https://www.ncbi.nlm.nih.gov/nuccore/QJQ80442.1&doptcmdl=GenBank) Product:hypothetical protein | PI:[*QEA10358.1*](https://www.ncbi.nlm.nih.gov/nuccore/QEA10358.1&doptcmdl=GenBank) Product:hypothetical protein | PI:[*YP_008239654.1*](https://www.ncbi.nlm.nih.gov/nuccore/YP_008239654.1&doptcmdl=GenBank) Product:hypothetical protein | PI:[*QFR58940.1*](https://www.ncbi.nlm.nih.gov/nuccore/QFR58940.1&doptcmdl=GenBank) Product:hypothetical protein | PI:[*AXQ70214.1*](https://www.ncbi.nlm.nih.gov/nuccore/AXQ70214.1&doptcmdl=GenBank) Product:hypothetical protein |
| PI:[*QVW27669.1*](https://www.ncbi.nlm.nih.gov/nuccore/QVW27669.1&doptcmdl=GenBank) Product:hypothetical protein | PI:[*QBJ04022.1*](https://www.ncbi.nlm.nih.gov/nuccore/QBJ04022.1&doptcmdl=GenBank) Product:hypothetical protein | PI:[*AZF89069.1*](https://www.ncbi.nlm.nih.gov/nuccore/AZF89069.1&doptcmdl=GenBank) Product:hypothetical protein | PI:[*QJQ80443.1*](https://www.ncbi.nlm.nih.gov/nuccore/QJQ80443.1&doptcmdl=GenBank) Product:hypothetical protein | PI:[*QEA10357.1*](https://www.ncbi.nlm.nih.gov/nuccore/QEA10357.1&doptcmdl=GenBank) Product:minor tail protein | PI:[*YP_008239653.1*](https://www.ncbi.nlm.nih.gov/nuccore/YP_008239653.1&doptcmdl=GenBank) Product:hypothetical protein | PI:[*QFR58939.1*](https://www.ncbi.nlm.nih.gov/nuccore/QFR58939.1&doptcmdl=GenBank) Product:DUF1833 domain-containing protein | PI:[*AXQ70213.1*](https://www.ncbi.nlm.nih.gov/nuccore/AXQ70213.1&doptcmdl=GenBank) Product:hypothetical protein |
| PI:[*QVW27670.1*](https://www.ncbi.nlm.nih.gov/nuccore/QVW27670.1&doptcmdl=GenBank) Product:hypothetical protein | PI:[*QBJ04023.1*](https://www.ncbi.nlm.nih.gov/nuccore/QBJ04023.1&doptcmdl=GenBank) Product:hypothetical protein | PI:[*AZF89070.1*](https://www.ncbi.nlm.nih.gov/nuccore/AZF89070.1&doptcmdl=GenBank) Product:hypothetical protein | PI:[*QJQ80444.1*](https://www.ncbi.nlm.nih.gov/nuccore/QJQ80444.1&doptcmdl=GenBank) Product:hypothetical protein | PI:[*QEA10356.1*](https://www.ncbi.nlm.nih.gov/nuccore/QEA10356.1&doptcmdl=GenBank) Product:hypothetical protein | PI:[*YP_008239652.1*](https://www.ncbi.nlm.nih.gov/nuccore/YP_008239652.1&doptcmdl=GenBank) Product:hypothetical protein | PI:[*QFR58938.1*](https://www.ncbi.nlm.nih.gov/nuccore/QFR58938.1&doptcmdl=GenBank) Product:hypothetical protein | PI:[*AXQ70212.1*](https://www.ncbi.nlm.nih.gov/nuccore/AXQ70212.1&doptcmdl=GenBank) Product:hypothetical protein |
| PI:[*QVW27671.1*](https://www.ncbi.nlm.nih.gov/nuccore/QVW27671.1&doptcmdl=GenBank) Product:tail length tape measure-related protein | PI:[*QBJ04024.1*](https://www.ncbi.nlm.nih.gov/nuccore/QBJ04024.1&doptcmdl=GenBank) Product:tail length tape measure-related protein | PI:[*AZF89071.1*](https://www.ncbi.nlm.nih.gov/nuccore/AZF89071.1&doptcmdl=GenBank) Product:tail length tape-measure protein 1 | PI:[*QJQ80445.1*](https://www.ncbi.nlm.nih.gov/nuccore/QJQ80445.1&doptcmdl=GenBank) Product:tail tape measure | PI:[*QEA10355.1*](https://www.ncbi.nlm.nih.gov/nuccore/QEA10355.1&doptcmdl=GenBank) Product:tape measure protein | PI:[*YP_008239651.1*](https://www.ncbi.nlm.nih.gov/nuccore/YP_008239651.1&doptcmdl=GenBank) Product:tail length tape measure-related protein | PI:[*QFR58937.1*](https://www.ncbi.nlm.nih.gov/nuccore/QFR58937.1&doptcmdl=GenBank) Product:tail length tape-measure protein | PI:[*AXQ70211.1*](https://www.ncbi.nlm.nih.gov/nuccore/AXQ70211.1&doptcmdl=GenBank) Product:tail length tape-measure protein |
| PI:[*QVW27672.1*](https://www.ncbi.nlm.nih.gov/nuccore/QVW27672.1&doptcmdl=GenBank) Product:putative tail assembly chaperone | PI:[*QBJ04026.1*](https://www.ncbi.nlm.nih.gov/nuccore/QBJ04026.1&doptcmdl=GenBank) Product:putative tail assembly chaperone | PI:[*AZF89072.1*](https://www.ncbi.nlm.nih.gov/nuccore/AZF89072.1&doptcmdl=GenBank) Product:hypothetical protein | PI:[*QJQ80447.1*](https://www.ncbi.nlm.nih.gov/nuccore/QJQ80447.1&doptcmdl=GenBank) Product:hypothetical protein | PI:[*QEA10354.1*](https://www.ncbi.nlm.nih.gov/nuccore/QEA10354.1&doptcmdl=GenBank) Product:tail assembly chaperone | PI:[*YP_008239649.1*](https://www.ncbi.nlm.nih.gov/nuccore/YP_008239649.1&doptcmdl=GenBank) Product:hypothetical protein | PI:[*QFR58935.1*](https://www.ncbi.nlm.nih.gov/nuccore/QFR58935.1&doptcmdl=GenBank) Product:hypothetical protein | PI:[*AXQ70209.1*](https://www.ncbi.nlm.nih.gov/nuccore/AXQ70209.1&doptcmdl=GenBank) Product:hypothetical protein |
| PI:[*QVW27672.1*](https://www.ncbi.nlm.nih.gov/nuccore/QVW27672.1&doptcmdl=GenBank) Product:putative tail assembly chaperone | PI:[*QBJ04026.1*](https://www.ncbi.nlm.nih.gov/nuccore/QBJ04026.1&doptcmdl=GenBank) Product:putative tail assembly chaperone | PI:[*AZF89072.1*](https://www.ncbi.nlm.nih.gov/nuccore/AZF89072.1&doptcmdl=GenBank) Product:hypothetical protein | PI:[*QJQ80447.1*](https://www.ncbi.nlm.nih.gov/nuccore/QJQ80447.1&doptcmdl=GenBank) Product:hypothetical protein | PI:[*QEA10353.1*](https://www.ncbi.nlm.nih.gov/nuccore/QEA10353.1&doptcmdl=GenBank) Product:tail assembly chaperone | PI:[*YP_008239649.1*](https://www.ncbi.nlm.nih.gov/nuccore/YP_008239649.1&doptcmdl=GenBank) Product:hypothetical protein | PI:[*QFR58935.1*](https://www.ncbi.nlm.nih.gov/nuccore/QFR58935.1&doptcmdl=GenBank) Product:hypothetical protein | PI:[*AXQ70209.1*](https://www.ncbi.nlm.nih.gov/nuccore/AXQ70209.1&doptcmdl=GenBank) Product:hypothetical protein |
| PI:[*QVW27674.1*](https://www.ncbi.nlm.nih.gov/nuccore/QVW27674.1&doptcmdl=GenBank) Product:putative tail protein | PI:[*QBJ04028.1*](https://www.ncbi.nlm.nih.gov/nuccore/QBJ04028.1&doptcmdl=GenBank) Product:putative tail protein | PI:[*AZF89074.1*](https://www.ncbi.nlm.nih.gov/nuccore/AZF89074.1&doptcmdl=GenBank) Product:hypothetical protein | PI:[*QJQ80449.1*](https://www.ncbi.nlm.nih.gov/nuccore/QJQ80449.1&doptcmdl=GenBank) Product:tail protein | PI:[*QEA10351.1*](https://www.ncbi.nlm.nih.gov/nuccore/QEA10351.1&doptcmdl=GenBank) Product:tail protein | PI:[*YP_008239647.1*](https://www.ncbi.nlm.nih.gov/nuccore/YP_008239647.1&doptcmdl=GenBank) Product:putative tail protein | PI:[*QFR58932.1*](https://www.ncbi.nlm.nih.gov/nuccore/QFR58932.1&doptcmdl=GenBank) Product:tail protein | PI:[*AXQ70206.1*](https://www.ncbi.nlm.nih.gov/nuccore/AXQ70206.1&doptcmdl=GenBank) Product:tail protein |
| PI:[*QVW27675.1*](https://www.ncbi.nlm.nih.gov/nuccore/QVW27675.1&doptcmdl=GenBank) Product:hypothetical protein | PI:[*QBJ04029.1*](https://www.ncbi.nlm.nih.gov/nuccore/QBJ04029.1&doptcmdl=GenBank) Product:hypothetical protein | PI:[*AZF89075.1*](https://www.ncbi.nlm.nih.gov/nuccore/AZF89075.1&doptcmdl=GenBank) Product:hypothetical protein | PI:[*QJQ80450.1*](https://www.ncbi.nlm.nih.gov/nuccore/QJQ80450.1&doptcmdl=GenBank) Product:structural protein | PI:[*QEA10350.1*](https://www.ncbi.nlm.nih.gov/nuccore/QEA10350.1&doptcmdl=GenBank) Product:hypothetical protein | PI:[*YP_008239646.1*](https://www.ncbi.nlm.nih.gov/nuccore/YP_008239646.1&doptcmdl=GenBank) Product:hypothetical protein | PI:[*QFR58931.1*](https://www.ncbi.nlm.nih.gov/nuccore/QFR58931.1&doptcmdl=GenBank) Product:hypothetical protein | PI:[*AXQ70205.1*](https://www.ncbi.nlm.nih.gov/nuccore/AXQ70205.1&doptcmdl=GenBank) Product:hypothetical protein |
| PI:[*QVW27676.1*](https://www.ncbi.nlm.nih.gov/nuccore/QVW27676.1&doptcmdl=GenBank) Product:putative tail protein | PI:[*QBJ04030.1*](https://www.ncbi.nlm.nih.gov/nuccore/QBJ04030.1&doptcmdl=GenBank) Product:putative tail protein | PI:[*AZF89076.1*](https://www.ncbi.nlm.nih.gov/nuccore/AZF89076.1&doptcmdl=GenBank) Product:hypothetical protein | PI:[*QJQ80451.1*](https://www.ncbi.nlm.nih.gov/nuccore/QJQ80451.1&doptcmdl=GenBank) Product:tail protein | PI:[*QEA10349.1*](https://www.ncbi.nlm.nih.gov/nuccore/QEA10349.1&doptcmdl=GenBank) Product:putative minor tail component | PI:[*YP_008239645.1*](https://www.ncbi.nlm.nih.gov/nuccore/YP_008239645.1&doptcmdl=GenBank) Product:hypothetical protein | PI:[*QFR58930.1*](https://www.ncbi.nlm.nih.gov/nuccore/QFR58930.1&doptcmdl=GenBank) Product:hypothetical protein | PI:[*AXQ70204.1*](https://www.ncbi.nlm.nih.gov/nuccore/AXQ70204.1&doptcmdl=GenBank) Product:hypothetical protein |
| PI:[*QVW27677.1*](https://www.ncbi.nlm.nih.gov/nuccore/QVW27677.1&doptcmdl=GenBank) Product:putative tail protein | PI:[*QBJ04031.1*](https://www.ncbi.nlm.nih.gov/nuccore/QBJ04031.1&doptcmdl=GenBank) Product:putative tail protein | PI:[*AZF89077.1*](https://www.ncbi.nlm.nih.gov/nuccore/AZF89077.1&doptcmdl=GenBank) Product:hypothetical protein | PI:[*QJQ80452.1*](https://www.ncbi.nlm.nih.gov/nuccore/QJQ80452.1&doptcmdl=GenBank) Product:tail protein | PI:[*QEA10348.1*](https://www.ncbi.nlm.nih.gov/nuccore/QEA10348.1&doptcmdl=GenBank) Product:putative minor tail protein | PI:[*YP_008239644.1*](https://www.ncbi.nlm.nih.gov/nuccore/YP_008239644.1&doptcmdl=GenBank) Product:hypothetical protein | PI:[*QFR58929.1*](https://www.ncbi.nlm.nih.gov/nuccore/QFR58929.1&doptcmdl=GenBank) Product:hypothetical protein | PI:[*AXQ70203.1*](https://www.ncbi.nlm.nih.gov/nuccore/AXQ70203.1&doptcmdl=GenBank) Product:hypothetical protein |
| PI:[*QVW27678.1*](https://www.ncbi.nlm.nih.gov/nuccore/QVW27678.1&doptcmdl=GenBank) Product:hypothetical protein | PI:[*QBJ04032.1*](https://www.ncbi.nlm.nih.gov/nuccore/QBJ04032.1&doptcmdl=GenBank) Product:hypothetical protein | PI:[*AZF89078.1*](https://www.ncbi.nlm.nih.gov/nuccore/AZF89078.1&doptcmdl=GenBank) Product:hypothetical protein | PI:[*QJQ80453.1*](https://www.ncbi.nlm.nih.gov/nuccore/QJQ80453.1&doptcmdl=GenBank) Product:neck protein | PI:[*QGZ04766.1*](https://www.ncbi.nlm.nih.gov/nuccore/QGZ04766.1&doptcmdl=GenBank) Product:hypothetical protein | PI:[*YP_008239643.1*](https://www.ncbi.nlm.nih.gov/nuccore/YP_008239643.1&doptcmdl=GenBank) Product:hypothetical protein | PI:[*QFR58928.1*](https://www.ncbi.nlm.nih.gov/nuccore/QFR58928.1&doptcmdl=GenBank) Product:hypothetical protein | PI:[*AXQ70202.1*](https://www.ncbi.nlm.nih.gov/nuccore/AXQ70202.1&doptcmdl=GenBank) Product:hypothetical protein |
| PI:[*QVW27679.1*](https://www.ncbi.nlm.nih.gov/nuccore/QVW27679.1&doptcmdl=GenBank) Product:hypothetical protein | PI:[*QBJ04033.1*](https://www.ncbi.nlm.nih.gov/nuccore/QBJ04033.1&doptcmdl=GenBank) Product:hypothetical protein | PI:[*AZF89079.1*](https://www.ncbi.nlm.nih.gov/nuccore/AZF89079.1&doptcmdl=GenBank) Product:hypothetical protein | PI:[*QJQ80454.1*](https://www.ncbi.nlm.nih.gov/nuccore/QJQ80454.1&doptcmdl=GenBank) Product:head-tail joining protein | PI:[*QEA10345.1*](https://www.ncbi.nlm.nih.gov/nuccore/QEA10345.1&doptcmdl=GenBank) Product:head-to-tail connector complex protein | PI:[*YP_008239642.1*](https://www.ncbi.nlm.nih.gov/nuccore/YP_008239642.1&doptcmdl=GenBank) Product:hypothetical protein | PI:[*QFR58927.1*](https://www.ncbi.nlm.nih.gov/nuccore/QFR58927.1&doptcmdl=GenBank) Product:hypothetical protein | PI:[*AXQ70201.1*](https://www.ncbi.nlm.nih.gov/nuccore/AXQ70201.1&doptcmdl=GenBank) Product:hypothetical protein |
| PI:[*QVW27680.1*](https://www.ncbi.nlm.nih.gov/nuccore/QVW27680.1&doptcmdl=GenBank) Product:hypothetical protein | PI:[*QBJ04034.1*](https://www.ncbi.nlm.nih.gov/nuccore/QBJ04034.1&doptcmdl=GenBank) Product:hypothetical protein | PI:[*AZF89080.1*](https://www.ncbi.nlm.nih.gov/nuccore/AZF89080.1&doptcmdl=GenBank) Product:hypothetical protein | PI:[*QJQ80455.1*](https://www.ncbi.nlm.nih.gov/nuccore/QJQ80455.1&doptcmdl=GenBank) Product:hypothetical protein | PI:[*QEA10344.1*](https://www.ncbi.nlm.nih.gov/nuccore/QEA10344.1&doptcmdl=GenBank) Product:hypothetical protein | PI:[*YP_008239641.1*](https://www.ncbi.nlm.nih.gov/nuccore/YP_008239641.1&doptcmdl=GenBank) Product:hypothetical protein | PI:[*QFR58926.1*](https://www.ncbi.nlm.nih.gov/nuccore/QFR58926.1&doptcmdl=GenBank) Product:hypothetical protein | PI:[*AXQ70200.1*](https://www.ncbi.nlm.nih.gov/nuccore/AXQ70200.1&doptcmdl=GenBank) Product:hypothetical protein |
| PI:[*QVW27681.1*](https://www.ncbi.nlm.nih.gov/nuccore/QVW27681.1&doptcmdl=GenBank) Product:putative tail protein | PI:[*QBJ04035.1*](https://www.ncbi.nlm.nih.gov/nuccore/QBJ04035.1&doptcmdl=GenBank) Product:putative tail protein | PI:[*AZF89081.1*](https://www.ncbi.nlm.nih.gov/nuccore/AZF89081.1&doptcmdl=GenBank) Product:hypothetical protein | PI:[*QJQ80456.1*](https://www.ncbi.nlm.nih.gov/nuccore/QJQ80456.1&doptcmdl=GenBank) Product:tail protein | PI:[*QEA10343.1*](https://www.ncbi.nlm.nih.gov/nuccore/QEA10343.1&doptcmdl=GenBank) Product:decoration protein | PI:[*YP_008239640.1*](https://www.ncbi.nlm.nih.gov/nuccore/YP_008239640.1&doptcmdl=GenBank) Product:hypothetical protein | PI:[*QFR58925.1*](https://www.ncbi.nlm.nih.gov/nuccore/QFR58925.1&doptcmdl=GenBank) Product:neck whiskers protein | PI:[*AXQ70199.1*](https://www.ncbi.nlm.nih.gov/nuccore/AXQ70199.1&doptcmdl=GenBank) Product:neck whiskers protein |
| PI:[*QVW27683.1*](https://www.ncbi.nlm.nih.gov/nuccore/QVW27683.1&doptcmdl=GenBank) Product:major capsid protein | PI:[*QBJ04037.1*](https://www.ncbi.nlm.nih.gov/nuccore/QBJ04037.1&doptcmdl=GenBank) Product:major capsid protein | PI:[*AZF89083.1*](https://www.ncbi.nlm.nih.gov/nuccore/AZF89083.1&doptcmdl=GenBank) Product:capsid protein | PI:[*QJQ80458.1*](https://www.ncbi.nlm.nih.gov/nuccore/QJQ80458.1&doptcmdl=GenBank) Product:major capsid protein | PI:[*QEA10341.1*](https://www.ncbi.nlm.nih.gov/nuccore/QEA10341.1&doptcmdl=GenBank) Product:major capsid protein | PI:[*YP_008239638.1*](https://www.ncbi.nlm.nih.gov/nuccore/YP_008239638.1&doptcmdl=GenBank) Product:coat protein | PI:[*QFR58923.1*](https://www.ncbi.nlm.nih.gov/nuccore/QFR58923.1&doptcmdl=GenBank) Product:capsid protein | PI:[*AXQ70197.1*](https://www.ncbi.nlm.nih.gov/nuccore/AXQ70197.1&doptcmdl=GenBank) Product:capsid protein |
| PI:[*QVW27684.1*](https://www.ncbi.nlm.nih.gov/nuccore/QVW27684.1&doptcmdl=GenBank) Product:hypothetical protein | PI:[*QBJ04038.1*](https://www.ncbi.nlm.nih.gov/nuccore/QBJ04038.1&doptcmdl=GenBank) Product:hypothetical protein | PI:[*AZF89084.1*](https://www.ncbi.nlm.nih.gov/nuccore/AZF89084.1&doptcmdl=GenBank) Product:hypothetical protein | PI:[*QJQ80384.1*](https://www.ncbi.nlm.nih.gov/nuccore/QJQ80384.1&doptcmdl=GenBank) Product:hypothetical protein | PI:[*QEA10340.1*](https://www.ncbi.nlm.nih.gov/nuccore/QEA10340.1&doptcmdl=GenBank) Product:scaffold protein | PI:[*YP_008239637.1*](https://www.ncbi.nlm.nih.gov/nuccore/YP_008239637.1&doptcmdl=GenBank) Product:hypothetical protein | PI:[*QFR58922.1*](https://www.ncbi.nlm.nih.gov/nuccore/QFR58922.1&doptcmdl=GenBank) Product:hypothetical protein | PI:[*AXQ70196.1*](https://www.ncbi.nlm.nih.gov/nuccore/AXQ70196.1&doptcmdl=GenBank) Product:hypothetical protein |
| PI:[*QVW27686.1*](https://www.ncbi.nlm.nih.gov/nuccore/QVW27686.1&doptcmdl=GenBank) Product:hypothetical protein | PI:[*QBJ04041.1*](https://www.ncbi.nlm.nih.gov/nuccore/QBJ04041.1&doptcmdl=GenBank) Product:hypothetical protein | PI:[*AZF89087.1*](https://www.ncbi.nlm.nih.gov/nuccore/AZF89087.1&doptcmdl=GenBank) Product:hypothetical protein | PI:[*QJQ80386.1*](https://www.ncbi.nlm.nih.gov/nuccore/QJQ80386.1&doptcmdl=GenBank) Product:hypothetical protein | PI:[*QEA10337.1*](https://www.ncbi.nlm.nih.gov/nuccore/QEA10337.1&doptcmdl=GenBank) Product:i-spanin | PI:[*YP_008239634.1*](https://www.ncbi.nlm.nih.gov/nuccore/YP_008239634.1&doptcmdl=GenBank) Product:hypothetical protein | PI:[*QFR58919.1*](https://www.ncbi.nlm.nih.gov/nuccore/QFR58919.1&doptcmdl=GenBank) Product:hypothetical protein | PI:[*AXQ70193.1*](https://www.ncbi.nlm.nih.gov/nuccore/AXQ70193.1&doptcmdl=GenBank) Product:spanin |
| PI:[*QVW27687.1*](https://www.ncbi.nlm.nih.gov/nuccore/QVW27687.1&doptcmdl=GenBank) Product:putative tail protein | PI:[*QBJ04043.1*](https://www.ncbi.nlm.nih.gov/nuccore/QBJ04043.1&doptcmdl=GenBank) Product:putative tail protein | PI:[*AZF89089.1*](https://www.ncbi.nlm.nih.gov/nuccore/AZF89089.1&doptcmdl=GenBank) Product:hypothetical protein | PI:[*QJQ80389.1*](https://www.ncbi.nlm.nih.gov/nuccore/QJQ80389.1&doptcmdl=GenBank) Product:fibritin protein | PI:[*QEA10335.1*](https://www.ncbi.nlm.nih.gov/nuccore/QEA10335.1&doptcmdl=GenBank) Product:hypothetical protein | PI:[*YP_008239632.1*](https://www.ncbi.nlm.nih.gov/nuccore/YP_008239632.1&doptcmdl=GenBank) Product:hypothetical protein | PI:[*QFR58917.1*](https://www.ncbi.nlm.nih.gov/nuccore/QFR58917.1&doptcmdl=GenBank) Product:hypothetical protein | PI:[*AXQ70190.1*](https://www.ncbi.nlm.nih.gov/nuccore/AXQ70190.1&doptcmdl=GenBank) Product:hypothetical protein |
| PI:[*QVW27688.1*](https://www.ncbi.nlm.nih.gov/nuccore/QVW27688.1&doptcmdl=GenBank) Product:hypothetical protein | PI:[*QBJ04044.1*](https://www.ncbi.nlm.nih.gov/nuccore/QBJ04044.1&doptcmdl=GenBank) Product:structural protein | PI:[*AZF89090.1*](https://www.ncbi.nlm.nih.gov/nuccore/AZF89090.1&doptcmdl=GenBank) Product:hypothetical protein | PI:[*QJQ80390.1*](https://www.ncbi.nlm.nih.gov/nuccore/QJQ80390.1&doptcmdl=GenBank) Product:putative head protein | PI:[*QEA10334.1*](https://www.ncbi.nlm.nih.gov/nuccore/QEA10334.1&doptcmdl=GenBank) Product:head morphogenesis protein | PI:[*YP_008239631.1*](https://www.ncbi.nlm.nih.gov/nuccore/YP_008239631.1&doptcmdl=GenBank) Product:head morphogenesis protein | PI:[*QFR58916.1*](https://www.ncbi.nlm.nih.gov/nuccore/QFR58916.1&doptcmdl=GenBank) Product:head morphogenesis protein | PI:[*AXQ70189.1*](https://www.ncbi.nlm.nih.gov/nuccore/AXQ70189.1&doptcmdl=GenBank) Product:head morphogenesis protein |
| PI:[*QVW27689.1*](https://www.ncbi.nlm.nih.gov/nuccore/QVW27689.1&doptcmdl=GenBank) Product:structural protein | PI:[*QBJ04045.1*](https://www.ncbi.nlm.nih.gov/nuccore/QBJ04045.1&doptcmdl=GenBank) Product:structural protein | PI:[*AZF89091.1*](https://www.ncbi.nlm.nih.gov/nuccore/AZF89091.1&doptcmdl=GenBank) Product:62kDa structural protein | PI:[*QJQ80392.1*](https://www.ncbi.nlm.nih.gov/nuccore/QJQ80392.1&doptcmdl=GenBank) Product:62kDa structural protein | PI:[*QEA10333.1*](https://www.ncbi.nlm.nih.gov/nuccore/QEA10333.1&doptcmdl=GenBank) Product:portal protein | PI:[*YP_008239630.1*](https://www.ncbi.nlm.nih.gov/nuccore/YP_008239630.1&doptcmdl=GenBank) Product:phage structural protein | PI:[*QFR58915.1*](https://www.ncbi.nlm.nih.gov/nuccore/QFR58915.1&doptcmdl=GenBank) Product:DUF4055 domain-containing protein | PI:[*AXQ70188.1*](https://www.ncbi.nlm.nih.gov/nuccore/AXQ70188.1&doptcmdl=GenBank) Product:DUF4055 domain-containing protein |
| PI:[*QVW27690.1*](https://www.ncbi.nlm.nih.gov/nuccore/QVW27690.1&doptcmdl=GenBank) Product:terminase large subunit | PI:[*QBJ04046.1*](https://www.ncbi.nlm.nih.gov/nuccore/QBJ04046.1&doptcmdl=GenBank) Product:terminase large subunit | PI:[*AZF89092.1*](https://www.ncbi.nlm.nih.gov/nuccore/AZF89092.1&doptcmdl=GenBank) Product:putative terminase | PI:[*QJQ80393.1*](https://www.ncbi.nlm.nih.gov/nuccore/QJQ80393.1&doptcmdl=GenBank) Product:terminase large subunit | PI:[*QEA10330.1*](https://www.ncbi.nlm.nih.gov/nuccore/QEA10330.1&doptcmdl=GenBank) Product:terminase large subunit | PI:[*YP_008239629.1*](https://www.ncbi.nlm.nih.gov/nuccore/YP_008239629.1&doptcmdl=GenBank) Product:hypothetical protein | PI:[*QFR58914.1*](https://www.ncbi.nlm.nih.gov/nuccore/QFR58914.1&doptcmdl=GenBank) Product:terminase large subunit | PI:[*AXQ70187.1*](https://www.ncbi.nlm.nih.gov/nuccore/AXQ70187.1&doptcmdl=GenBank) Product:terminase large subunit |
| PI:[*QVW27691.1*](https://www.ncbi.nlm.nih.gov/nuccore/QVW27691.1&doptcmdl=GenBank) Product:terminase small subunit | PI:[*QBJ04047.1*](https://www.ncbi.nlm.nih.gov/nuccore/QBJ04047.1&doptcmdl=GenBank) Product:terminase small subunit | PI:[*AZF89093.1*](https://www.ncbi.nlm.nih.gov/nuccore/AZF89093.1&doptcmdl=GenBank) Product:hypothetical protein | PI:[*QJQ80394.1*](https://www.ncbi.nlm.nih.gov/nuccore/QJQ80394.1&doptcmdl=GenBank) Product:hypothetical protein | PI:[*QEA10329.2*](https://www.ncbi.nlm.nih.gov/nuccore/QEA10329.2&doptcmdl=GenBank) Product:terminase small subunit | PI:[*YP_008239628.1*](https://www.ncbi.nlm.nih.gov/nuccore/YP_008239628.1&doptcmdl=GenBank) Product:hypothetical protein | PI:[*QFR58913.1*](https://www.ncbi.nlm.nih.gov/nuccore/QFR58913.1&doptcmdl=GenBank) Product:hypothetical protein | PI:[*AXQ70186.1*](https://www.ncbi.nlm.nih.gov/nuccore/AXQ70186.1&doptcmdl=GenBank) Product:terminase small subunit |
| PI:[*QVW27692.1*](https://www.ncbi.nlm.nih.gov/nuccore/QVW27692.1&doptcmdl=GenBank) Product:hypothetical protein | PI:[*QBJ04048.1*](https://www.ncbi.nlm.nih.gov/nuccore/QBJ04048.1&doptcmdl=GenBank) Product:hypothetical protein | PI:[*AZF89095.1*](https://www.ncbi.nlm.nih.gov/nuccore/AZF89095.1&doptcmdl=GenBank) Product:hypothetical protein | PI:[*QJQ80396.1*](https://www.ncbi.nlm.nih.gov/nuccore/QJQ80396.1&doptcmdl=GenBank) Product:putative NinH-like protein | PI:[*QEA10411.1*](https://www.ncbi.nlm.nih.gov/nuccore/QEA10411.1&doptcmdl=GenBank) Product:hypothetical protein | PI:[*YP_008239626.1*](https://www.ncbi.nlm.nih.gov/nuccore/YP_008239626.1&doptcmdl=GenBank) Product:hypothetical protein | PI:[*QFR58984.1*](https://www.ncbi.nlm.nih.gov/nuccore/QFR58984.1&doptcmdl=GenBank) Product:hypothetical protein | PI:[*AXQ70257.1*](https://www.ncbi.nlm.nih.gov/nuccore/AXQ70257.1&doptcmdl=GenBank) Product:hypothetical protein |
| PI:[*QVW27693.1*](https://www.ncbi.nlm.nih.gov/nuccore/QVW27693.1&doptcmdl=GenBank) Product:hypothetical protein | PI:[*QBJ04049.1*](https://www.ncbi.nlm.nih.gov/nuccore/QBJ04049.1&doptcmdl=GenBank) Product:hypothetical protein | PI:[*AZF89096.1*](https://www.ncbi.nlm.nih.gov/nuccore/AZF89096.1&doptcmdl=GenBank) Product:hypothetical protein | PI:[*QJQ80397.1*](https://www.ncbi.nlm.nih.gov/nuccore/QJQ80397.1&doptcmdl=GenBank) Product:Nin protein | PI:[*QEA10407.1*](https://www.ncbi.nlm.nih.gov/nuccore/QEA10407.1&doptcmdl=GenBank) Product:hypothetical protein | PI:[*YP_008239624.1*](https://www.ncbi.nlm.nih.gov/nuccore/YP_008239624.1&doptcmdl=GenBank) Product:hypothetical protein | PI:[*QFR58981.1*](https://www.ncbi.nlm.nih.gov/nuccore/QFR58981.1&doptcmdl=GenBank) Product:hypothetical protein | PI:[*AXQ70254.1*](https://www.ncbi.nlm.nih.gov/nuccore/AXQ70254.1&doptcmdl=GenBank) Product:hypothetical protein |
| PI:[*QVW27694.1*](https://www.ncbi.nlm.nih.gov/nuccore/QVW27694.1&doptcmdl=GenBank) Product:hypothetical protein | PI:[*QBJ04050.1*](https://www.ncbi.nlm.nih.gov/nuccore/QBJ04050.1&doptcmdl=GenBank) Product:hypothetical protein | PI:[*AZF89097.1*](https://www.ncbi.nlm.nih.gov/nuccore/AZF89097.1&doptcmdl=GenBank) Product:hypothetical protein | PI:[*QJQ80398.1*](https://www.ncbi.nlm.nih.gov/nuccore/QJQ80398.1&doptcmdl=GenBank) Product:hypothetical protein | PI:[*QEA10404.1*](https://www.ncbi.nlm.nih.gov/nuccore/QEA10404.1&doptcmdl=GenBank) Product:hypothetical protein | PI:[*YP_008239623.1*](https://www.ncbi.nlm.nih.gov/nuccore/YP_008239623.1&doptcmdl=GenBank) Product:hypothetical protein | PI:[*QFR58979.1*](https://www.ncbi.nlm.nih.gov/nuccore/QFR58979.1&doptcmdl=GenBank) Product:DUF2737 domain-containing protein | PI:[*AXQ70252.1*](https://www.ncbi.nlm.nih.gov/nuccore/AXQ70252.1&doptcmdl=GenBank) Product:hypothetical protein |
